# Cell cycle arrest and apoptotic studies of *Terminalia chebula* against MCF-7 breast cancer cell line: an *in vitro* and *in silico* approach

**DOI:** 10.3389/fonc.2023.1221275

**Published:** 2023-08-29

**Authors:** Pruthvish Reddy, Sushma Pradeep, Gopinath S. M., Chandan Dharmashekar, Disha G., Sai Chakith M. R., Chandrashekar Srinivasa, Ali A. Shati, Mohammad Y. Alfaifi, Serag Eldin I. Elbehairi, Raghu Ram Achar, Ekaterina Silina, Victor Stupin, Natalia Manturova, Chandan Shivamallu, Shiva Prasad Kollur

**Affiliations:** ^1^ Department of Biotechnology, Acharya Institute of Technology, Bengaluru, Karnataka, India; ^2^ Department of Biotechnology and Bioinformatics, School of Life Sciences, JSS Academy of Higher Education and Research, Mysuru, Karnataka, India; ^3^ Department of Neurochemistry, National Institute of Mental Health and Neiurosciences, Bangalore, Karnataka, India; ^4^ Department of Pharmacology, JSS Medical College, JSS Academy of Higher Education and Research, Mysuru, Karnataka, India; ^5^ Department of Studies in Biotechnology, Davangere University, Davangere, Karnataka, India; ^6^ Biology Department, Faculty of Science, King Khalid University, Abha, Saudi Arabia; ^7^ Division of Biochemistry, School of Life Sciences, JSS Academy of Higher Education and Research, Mysuru, Karnataka, India; ^8^ Department of Hospital Surgery, N.I. Pirogov Russian National Research Medical University (RNRMU), Moscow, Russia; ^9^ School of Physical Sciences, Amrita Vishwa Vidyapeetham, Mysuru, Karnataka, India

**Keywords:** breast cancer, apoptosis, phytocompounds, EGFR, hemolysis

## Abstract

Breast cancer is a leading cause of mortality in women, and alternative therapies with fewer side effects are actively being explored. Breast cancer is a significant global health concern, and conventional treatments like radiotherapy and chemotherapy often have side effects. Medicinal plant extracts offer a promising avenue for the development of effective and safe anticancer therapies. *Terminalia chebula*, a plant known for its medicinal properties, was selected for investigation in this study. We aimed to assess the antiproliferative effects of TCF extract on breast cancer cells and explore the potential role of saccharopine, a phytochemical found in TCF, as an anticancer agent. MCF7 breast cancer cell lines were exposed to TCF extract, and cell viability and apoptosis assays were performed to evaluate the antiproliferative and apoptogenic effects. Molecular docking studies were conducted to assess the binding affinity of saccharopine with EGFRs. Molecular dynamics simulations and binding energy calculations were employed to analyze the stability of the EGFR-saccharopine complex. The TCF extract exhibited significant antiproliferative effects on MCF7 breast cancer cells and induced apoptosis in a dose-dependent manner. Molecular docking analysis revealed that saccharopine demonstrated a higher binding affinity with EGFR compared to the reference compound (17b-estradiol). The subsequent MDS simulations indicated stable binding patterns and conformation of the EGFR-saccharopine complex, suggesting a potential role in inhibiting EGFR-mediated signaling pathways. The investigation of Terminalia chebula fruit extract and its phytochemical saccharopine has revealed promising antiproliferative effects and a strong binding affinity with EGFR. These findings provide a foundation for future research aimed at isolating saccharopine and conducting in vivo studies to evaluate its potential as a targeted therapy for breast cancer. The development of novel anticancer agents from plant sources holds great promise in advancing the field of oncology and improving treatment outcomes for breast cancer patients.

## 1 Introduction

Cancer is one of the deadliest illnesses worldwide. The global disease burden in 2018 was estimated to be 18.1 million new malignant cases (1). In India, there were approximately 2.25 million recorded cases and over 784,000 deaths in the year 2020 as per the National Institute of Disease Avoidance and Research.

Carcinoma, sarcoma, lymphoma, melanoma, and leukemia are the five major types of cancer, with carcinoma being the most extensively studied (2). Carcinomas predominantly affect organs such as the lungs, breasts, skin, glands, and pancreas. The treatment of cancer involves various approaches, including surgery, chemotherapy, hormonal therapy, radiation therapy, and immunotherapy (3). However, these treatments have certain limitations, including high costs, physical and mental side effects, frequent hospital visits, and avoidable complications. Cancer conditions can lead to an increased production of free radicals by the immune system, resulting in oxidative stress, impaired cell function, and a compromised immune state (4).

Breast cancer is the most common cancer among women and ranks second overall when considering both genders (5). Globally, over 1 million women are diagnosed with breast cancer annually, and more than 410,000 lose their lives to this disease, accounting for 14% of female cancer deaths. Furthermore, the prevalence of breast cancer is projected to increase by up to 5% per year in several non-industrialized countries (6). It is estimated that by 2030, 192,370 women will be diagnosed with breast cancer, and 40,170 women will succumb to the disease. In India, breast cancer is the second most common cancer after cervical cancer, contributing to 19% of the total cancer burden in women (7). Approximately 80,000 women are diagnosed with breast cancer, and 40,000 lose their lives to the disease annually in India (3).

Breast cancer is characterized by the abnormal growth of cancer cells in the epithelial cells lining the lobules or ducts of the breast (ductal epithelium). The primary tumor originates in the breast itself but can become invasive and spread beyond the breast to regional lymph nodes or other organs, leading to a systemic disease (8). Metastatic spread, which is the main cause of breast cancer-related deaths, is often facilitated by tumor cells utilizing lymphatic routes for early dissemination. Tumor cells that remain in the lymph nodes can proliferate and eventually breach the node’s capsule. Lymph nodes can also serve as a source from which tumor cells enter the bloodstream, spreading to distant organ sites such as the bones, liver, brain, and lungs (9).

India, known for its rich ecological diversity, has provided abundant resources, including plants, with potential therapeutic properties for various diseases. Plants are known to contain phytoconstituents that possess pharmacological qualities, such as anti-diabetic, anticancer, and cardioprotective effects. However, before identifying the biologically active compounds responsible for these properties, it is crucial to evaluate their antiproliferative characteristics. Once the anticancer properties of a plant have been established, dried forms of the plant can be used for patient treatment. However, it is necessary to isolate and concentrate the therapeutically significant molecules while eliminating any toxic compounds present (10).

*Terminalia chebula* Retzius (*T. chebula* Retz.), also known as black Myroblans, is a plant from the *Terminalia* genus that is widely distributed in tropical regions worldwide. It has been traditionally used in folk medicine and studied for its various properties, including homeostatic, antitussive, laxative, diuretic, and cardiotonic effects. Several *Terminalia* species, including *T. chebula*, have been investigated for their potential in treating cancer. Fruit extracts from *T. chebula* have shown memory-enhancing effects by improving neurotransmission in the central cholinergic system, which may be beneficial for Alzheimer’s disease patients. Alcoholic root extracts from the plant have demonstrated local analgesic properties and can be used in oral surgeries at low concentrations. Furthermore, these extracts have shown potential in treating depression by interacting with dopamine and adrenergic receptors, leading to increased levels of noradrenaline and dopamine in the rat brain (11). Owing to its remarkable healing abilities, *T. chebula* is often referred to as the “king of medicines” in Ayurveda. It is believed to have broad-spectrum therapeutic effects, promoting tissue growth, and overall health while eliminating diseases and toxins from the body. *T. chebula* has been utilized in traditional formulations for its anti-diabetic, anti-inflammatory, laxative, antibacterial, antifungal, cardiotonic, diuretic, hyperlipidemic, jaundice, anti-helminthic, aphrodisiac, and restorative properties (12).

Nowadays, it is common to use bioinformatics tools to identify drugs for treating various diseases. One such tool is structure-based drug design, which allows for virtual screening of compounds to identify potential therapeutic candidates by examining their binding affinities with protein receptors before conducting *in vitro* and *in vivo* experiments. In this study, the performance of integrated computational approaches involving molecular docking (MD), molecular dynamics simulation (MDS), and binding energy calculations was evaluated during interaction with protein receptors to identify potential anti-carcinogenic properties of TCF extracts against breast cancer cell line. Cytotoxicity analysis was performed using MTT (3-(4,5-Dimethylthiazol-2-yl)-2,5-diphenyltetrazolium bromide) assay of the ethanolic extract of TCF conducted on the MCF7 breast cancer cell line, and the bioactive compounds of TCF were further evaluated for their anticancer properties using MD, MDS, and binding energy calculation studies. Epidermal growth factor receptor (EGFR), also known as ErbB1 or HER1, is a cell surface receptor that plays a crucial role in various cellular processes, including cell growth, proliferation, and survival. It belongs to the ErbB family of receptor tyrosine kinases and is expressed on the surface of many cell types, particularly in epithelial tissues. Activation of EGFR occurs upon binding of specific ligands, such as epidermal growth factor (EGF) or transforming growth factor alpha (TGF-α). This binding leads to receptor dimerization and autophosphorylation of tyrosine residues in the intracellular domain, initiating downstream signaling cascades. The activation of EGFR triggers various intracellular pathways, such as the Ras/MAPK pathway and the PI3K/Akt pathway, which regulate cell proliferation, survival, and differentiation. Dysregulation of EGFR signaling is associated with numerous diseases, including cancer, where overexpression or mutations in EGFR can lead to uncontrolled cell growth and tumor progression. Therefore, EGFR has become an important target for therapeutic interventions, and drugs that inhibit EGFR signaling, such as tyrosine kinase inhibitors and monoclonal antibodies, have been developed for the treatment of certain cancers. The study of EGFR and its signaling pathways continues to provide valuable insights into cellular biology and potential avenues for targeted therapies.

## 2 Materials and methods

The chemicals required for the study were obtained from Loba chemicals (Bangalore, India). Demineralized water was collected from an ELGA RO system and was used throughout the experiments (Elga Veolia, Lane End, UK). Breast cancer cells (MCF-7) were procured from the ATCC and cultured in Dulbecco’s Modified Eagle Medium (DMEM) with 10% fetal bovine serum (FBS), penicillin (100 IU/ml), and streptomycin (100 μg/ml) in 5% CO_2_ at 37°C until confluence.

### 2.1 Preparation of *T. chebula* fruit extract

The sample was ordered from the Amruth Kesari depot, and it was dried in the shade for 3 days in a row on hot, sunny days. After that, the sample was ground into powder. After that, it was weighed and kept in a cool, dark room. To begin the ethanol extraction process, a 10-g sample was weighed, and 50 ml of 100% ethanol was added one at a time. The sample was kept in a water bath with the temperature set at 50°C for 4 h. Whatman filter paper was used to filter the samples after the incubation period of 4 h. The filtrate was then held at 80°C to allow the ethanol to evaporate (ethanol has a boiling point of 78.3°C) and produce the concentrated dried plant extract that can be utilized right away in various dilutions to analyze the apoptosis of MCF7 cells (13).

### 2.2 Phytochemical screening of the prepared plant extract

The presence of many substances, including alkaloids, phenols, flavonoids, tannins, saponins, glycosides, and many more, was determined using a qualitative phytochemical assay. The plant extract was dissolved in ethanol at a concentration of 100 mg/ml. It was then kept for dissolving in vortex at 50°C for 10 min. Each phytochemical assay was then conducted using it (12). The below-described assays were performed.

a. Screening for Alkaloids (Dragendroff’s test)
  Twenty microliters of concentrated HCl was added to 200 µl of the plant extract and then mixed properly. To the obtained solution, 100 µl of Dragendroff’s reagent was added and then mixed well. Red precipitate appeared, indicating the presence of alkaloids (14).
b. Screening for carbohydrate (Molish’s test)
  A few drops of Molish’s reagent were added to 200 µl of plant extract taken in a test tube. After mixing properly, a few drops of concentrated HCl were added slowly. Formation of red or violet ring at the junction of layer in tube indicated the presence of carbohydrates (15).
c. Screening for tannins
  In 200 µl of extract, a few drops of 1% FeCl_3_ solution were added while stirring. Formation of green precipitate points the presence of tannins (16).
d. Screening for terpenoid (Salkowski s test)
  Two hundred microliters of chloroform and three to four drops of concentrated HCl were added to 200 µl of plant extract. It was then mixed well and kept for a few minutes. The formation of yellow precipitate indicated the presence of terpenoids (17).
e. Screening for glycoside (Liebermann’s test)
  In a glass test tube, 200 µl of plant extract was taken; later, chloroform and acetic acid were added and mixed. The mixture was then cooled in ice and concentrated HCl was added. The color change from violet to blue to green indicated the presence of glycosides (14).
f. Screening for steroid
  To 200 µl of plant extract, 200 µl of chloroform and concentrated HCl were added. Presence of red color on the top layer indicated the presence of steroids in the sample (15).
g. Screening for saponin
  Two hundred microliters of distilled water was added to 200 µl of plant extract solution. It was then shaken and warmed. Formation of stable foam indicated the presence of saponins (17).
h. Screening for flavonoids
  Two hundred microliters of 10% lead acetate was added to equal volume of plant extract solution. The appearance of yellow-colored precipitate indicated the presence of flavonoids (16).
i. Screening for amino acid (ninhydrin test)
  Two hundred microliters of the plant extract was mixed in 200 µl of 0.2% ninhydrin, which, upon reduction, resulted in a violet color, indicating the presence of amino acids (16).
j. Screening for mucilage
  Two hundred microliters of the plant extract was dissolved in 500 µl of absolute alcohol and then allowed to dry. The absence of precipitate solution indicated the presence of mucilage (16).
k. Screening for volatile oils
  Two hundred microliters of plant extract sample was mixed with dilute HCl. White precipitate indicated the presence of volatile oils (17).

### 2.3 Quantitative phytochemical analysis

#### 2.3.1 DPPH radical scavenging assay

Antioxidants scavenge DPPH (2,2-diphenylpicrylhydrazyl) radicals by donating an electron, resulting in the reduction of DPPH. Its decrease in absorbance at wavelength 517 nm can be used to measure how quickly the color changed from purple to yellow after reduction. Different concentrations (20, 40, 60, 80, and 100 μg/ml) of gallic acid and plant sample were added to multiple test tubes. Methanol was added for the volume to reach 500 μl. Five milliliters of a 0.1 mM DPPH methanolic solution was added to each test tube, which was then vortexed. For 20 min, the tubes were left to stand at room temperature. Methanol was utilized for the baseline correction, and the control was made in the same way as the sample but without any extract. At 517 nm, changes in the sample’s absorbance were detected (17). The inhibition percentage was used to express the radical scavenging activity. Gallic acid was used as standard. The relationship between concentration and percentage inhibition was plotted, and the IC50 value, the concentration needed to inhibit radicals by 50%, was calculated.

#### 2.3.2 ABTS radical scavenging assay

The (18) method was used to determine the ABTS [2,2′-azino-bis(3-ethylbenzothiazoline-6-sulfonic acid)] radical scavenging assay. The relative ability of antioxidants to squelch the radical cation ABTS^+^ served as the basis for the test. The ABTS decolorization assay involves the oxidation of ABTS with ammonium persulfate to produce the ABTS^+^ chromophore. Both hydrophilic and lipophilic molecules can use it. At 734 nm, the plant extract’s capacity to scavenge ABTS radical cation was determined. The reaction was started by adding 0.1 ml of diluted ABTS to 10 μl of variously concentrated aqueous extracts from the sample and 10 μl of methanol to act as the control, each of which had a different concentration. At 734 nm, the absorbance was measured (19).

### 2.4 MCF7 cell line preparation

The frozen MCF7 cell cryovials were taken from the liquid nitrogen storage and were thawed at a 37°C water bath by gentle shaking for less than 1 min until 70% of the culture is thawed. The vial was wiped with 70% ethanol to maintain aseptic conditions and then taken into the laminar air flow hood. Thawed cells were transferred into a 15-ml falcon tube and the required volume of incomplete DMEM media was added. Cell suspension was centrifuged at 11 rcf (*g*-value) for 5 min. The supernatant was discarded aseptically and DMEM medium was added to the cell pellet and resuspended in it. The number of viable cells was counted under the inverted microscope using Neubaur’s counting chamber after staining it with Trypan blue dye. The cell suspension was transferred to a 25-cm^2^ T flask and incubated at 37°C in a 5% CO_2_ incubator (20).

#### 2.4.1 Trypan blue viable cell counting method

No viable cells lose their membrane integrity; thus, trypan blue dye enters the dead cells, then they appear blue under the microscope. In contrast, viable cells that do not take up the dye appear white; 0.4% trypan blue solution was prepared by adding trypan blue in phosphate buffer saline, maintaining the pH at 7.4. Trypan blue solution and cell suspension were mixed in equal proportion. Cell suspension (20 µl) was loaded onto a hemacytometer and the cells were observed under low magnification. The number of viable cells and the total number of cells were counted (21).

#### 2.4.2 Hemocytometer

Total number of cells in a given cell suspension was determined by counting the number of cells in the four squares and then taking their average using the following formula (21):


$$Total\;number\;of\;cells/ml=Average\;no.\;of\;cells\times 10^{4}$$

##### 2.4.2.1 MTT cell cytotoxicity assay

Three rows of 96-well plates with an average cell density of 50,000 per well were seeded with cells on the first day and was kept overnight in a CO_2_ incubator at 37°C, while a stock solution of plant extract in DMSO with a 32 mg/ml concentration was created on the second day. It was spun for 5 min to remove any debris. Six different dilutions (10, 20, 40, 80, 160, and 320 μg/ml) of the plant extract were prepared in the entire DMEM medium using the stock solution. A 96-well plate seeded with medium was removed, and media with various plant extract dilutions were added. Complete media to the reference wells and 1% DMSO-treated media to the control wells were added. The culture plate was incubated overnight in a CO_2_ incubator at 37°C. The treated cells were examined using an inverted microscope on the third day. MTT powder in phosphate buffer saline (pH 7.2) at a concentration of 5 mg/10 ml was used to prepare MTT reagent. Media was removed and the 96-well plates were filled with 100 μl of MTT reagent followed by incubation for 3–4 h at 37°C. The obtained formazan was dissolved by adding 100 μl of pure DMSO after removing the MTT reagent. Microplate reader was used to measure the absorbance at 570 nm after thoroughly shaking the plate (22). Inhibition percentage was calculated by the following formula:


%Inhibition=100−(OD of sample/OD of control)×100

##### 2.4.2.2 Hemolysis assay

Blood (5 ml) was collected from healthy individuals, added to a vial containing EDTA, and mixed properly. The cells were centrifuged at 8 rcf for 10 min at 4°C. The supernatant plasma and white buffy layer were removed using a pipette while the collected erythrocytes were washed with 1×PBS thrice and the cells were diluted 10 times. Collected cells (100 µl) were added into 900 µl of 1×PBS. For positive control, 100 µl of 1% SDS was added to 50 µl of cells. For negative control, 100 µl of 1×PBS was added to 50 µl of cells. For treatment, 100 µl of 10 µg/ml and 15 µg/ml dilution of each sample was added to 50 µl of cells to different Eppendorf tubes and incubated at 37°C for 1 h. The reaction volume was adjusted to 1 ml by adding 850 µl of 1×PBS. Each reaction was centrifuged at 1 rcf for 5 min. Finally, 100 µl of supernatant of each reaction was transferred to the microtiter plate and measured for absorbance at 590 nm (23). Percentage hemolysis was calculated by the formula:


%Hemolysis=(Control−Sample)×100/Control

##### 2.4.2.3 G2M phase studies

MCF-7 cells were cultured in a six-well plate with 2 ml of complete medium, with a cell density of roughly 1 million per well. Cells were incubated in a CO_2_ incubator at 37°C for 24 h. The cells were treated with the samples at a concentration of 10 and 15 µg/ml, respectively, with 1% DMSO as control and incubated in a CO_2_ incubator at 37°C for 24 h. Trypsin treatment was used to separate the cells in each vial before collecting them. The cell suspension was centrifuged at 18 rcf for 5 min. Once the supernatant was carefully discarded, the cell pellet was thoroughly washed in 1× PBS. While blending the cell pellet, 200 ml of PBS and 1 ml of the fixing solution (70% EtOH) was added drop by drop. After that, immediately 1 ml of the fixing solution was added. The cell was maintained at 4°C for overnight fixation. Later, the cells were centrifuged at 125 rcf for 10 min at room temperature. The supernatant was removed with care, without disrupting the particle, and the pellet was washed twice in cold PBS. The cell pellet was dissolved in 500 µl of a propidium iodide (PI) solution containing 0.05 mg/ml of PI and RNaseA in PBS. FACS Calibur (BD Biosciences, San Jose, CA) was used to record the cells at different cell cycle stages (24).

##### 2.4.2.4 Apoptotic studies

In a six-well DMEM cell culture plate, 1 × 10^6^ MCF-7 cells were seeded per well and incubated overnight in a CO_2_ incubator at 37°C. The cells were treated with the supplied samples at concentrations of 10 and 15 µg/ml in DMEM basic medium while leaving one control untreated. This was then incubated overnight in a CO_2_ incubator at 37°C. The cells were collected from the well and placed in several vials with medium. Cell suspension was centrifuged at 71 rcf for 10 min, and the supernatant was carefully discarded without disrupting the cell pellet. Cells should be washed twice with cold PBS before being resuspended in 1 × PBS at a concentration of approximately 1 × 10^6^ cells/ml. A total of 1 × 10^5^ cells in 100 µl of cells are transferred to a 5-ml FACS tube (or 12 × 75 mm tube). To this, 5 µl each of PI and Annexin V was added. Cells were carefully mixed and left to incubate for 15 min at room temperature in the dark. Each tube was filled with 400 µl of 1 × binding buffer before being analyzed by flow cytometry as quickly as feasible (within an hour) (25). The sequential identification and refinement of a population of cells with the target molecule were visualized using fluorescence studies.

### 2.5 LDH assay

MCF7 cells were plated on 96-well cell culture plates at a density of 1 × 10^4^ cells per well, and they were then left to incubate for 24 h. Following medium changes and treatments with TCF concentrations of 10, 20, 40, and 80 µg/ml, the cells were cultivated for 24 h at 37°C. A cytoplasmic enzyme known as LDH (lactate dehydrogenase) was retained by healthy cells with intact plasma membranes but released by necrotic cells with damaged membranes. The Cytotoxicity Detection Kit was used to measure the released LDH in the media following incubation (26).

### 2.6 AO/EtBr staining assay

MCF7 cells were seeded on 96-well cell culture plates at a density of 1 × 10^4^ cells per well, and they were then left to incubate for 24 h. Both living and dead cells were stained by the necessary dye acridine orange. Ethidium bromide can only stain cells that have lost the integrity of their membranes. Live cells would all be the same color of green. In a microcentrifuge tube, 25 µl (approximately 1 × 10^5^ cells) of treated and untreated cells were collected individually and stained with 5 µl of AO-EtBr (acridine orange and ethidium bromide) for approximately 2 min before being gently mixed. A glass coverslip was placed over a microscopic slide containing 10 µl of cell suspension, and the slide was then studied using a fluorescence microscope with a fluorescein filter (27). (Depending on the type of cell, a higher or lower magnification may be preferred. Nuclear morphology ought to be apparent.)

### 2.7 ROS determination

In a six-well DMEM cell culture plate, 1 × 10^6^ MCF-7 cells were seeded per well and incubated overnight in a CO_2_ incubator at 37°C. The cell monolayer was washed with fresh media and cells were supplemented with fresh media and incubated for 2 h before proceeding with the treatment. DCFDA (2′-7′-dichlorodihydrofluorescein diacetate) solution at 25 µM was prepared in DMEM media and 100 µl of the solution was added to each well and incubated for 45 min in the above-mentioned conditions. DCFDA solution was removed carefully, and cell monolayer was briefly washed with 1×PBS once without affecting the cell monolayer. Cells were then treated with and without test compounds of various concentrations for 2 h, and NAC (N-acetyl-L-cysteine) at 5 mM was used as a positive control (ROS inhibitor). After 2 h of incubation, the cells were then treated with H_2_O_2_ at 500 µM and incubated for further for 2 h. The fluorescent signal was read at 485 nm.

### 2.8 *In silico* studies

#### 2.8.1 Protein and ligand preparation

**Figure 1:**
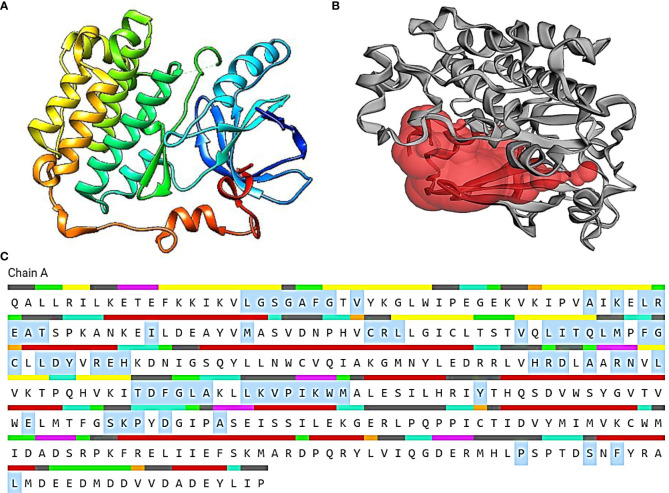
**(A)** The 3D representation of the 3W2S receptor. **(B)** The red pocket represents the binding pocket of the receptor. **(C)** The amino acid residues highlighted in gray specifies the amino acids present in the binding pocket of the 3W2S receptor.

The Protein Data Bank (PDB) tool (https://www.rcsb.org/) was used to retrieve the structure of EGFR receptor with PDB ID: 3W2S (27–29). After modeling the structure, the PDB coordinate file was submitted to the Procheck server. The most favored portions of the server plot, which is a graphical depiction of protein 3D structure, were confirmed. The protein model was confirmed, and the sections were identified by distinct colors. Furthermore, the binding sites of the modeled proteins were taken from the CASTp webserver (**Figure 1**) (30–33).

The IMPPAT database (https://cb.imsc.res.in/imppat/basicsearchauth) (34) was used to obtain the phytocompounds of TCF. This database includes more than 1,700 Indian medicinal plants with 1,100 therapeutic uses. The phytocompounds were identified based on their ADMET properties (35). The library of 20 molecules was made and the structure of the top five molecules that showed good and comparatively least binding affinity towards the selected proteins is depicted in **Table 1**. The study used 17β-estradiol as a reference compound because the estrogen receptor alpha is a significant biological target that mediates the development of breast cancer driven by this compound. The 3D and 2D structures of the compounds were obtained from the PubChem database (https://pubchem.ncbi.nlm.nih.gov/) in structure data file (SDF) format. The 2D structures were then converted into 3D coordinates and geometries using Open Babel, an open-source chemical toolbox. Subsequently, these structures were converted into PDB format (36, 37).

**Table 1:** The structures of the top five molecules and standard drug docked with the 3W2S receptor.

Sl No.	Name of the molecule	Binding energy in kcal/mol 3W2S	Structure of the molecule
**1**	Phthalamic acid	−8.4	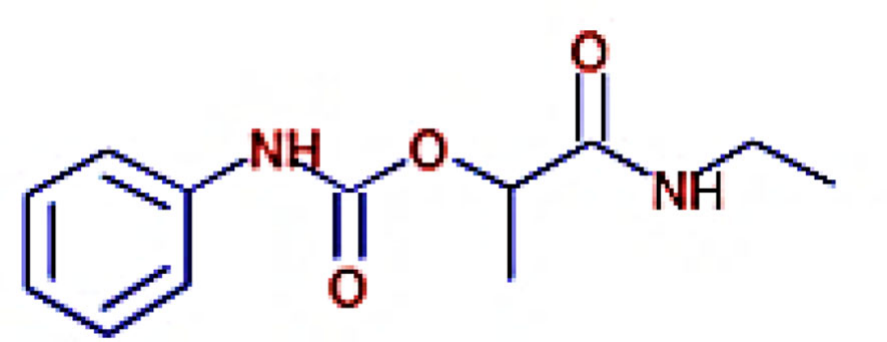
**2**	Saccharopine	−9.7	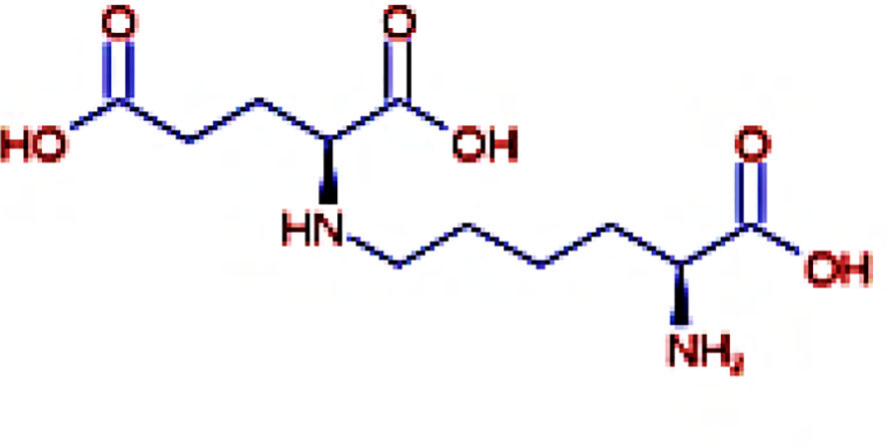
**3**	Mauritianin	−8.1	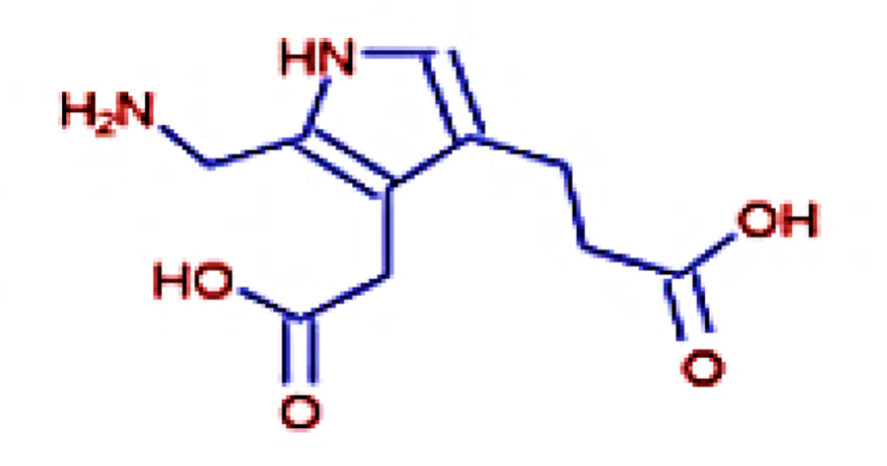
**4**	Carbetamide	−7.3	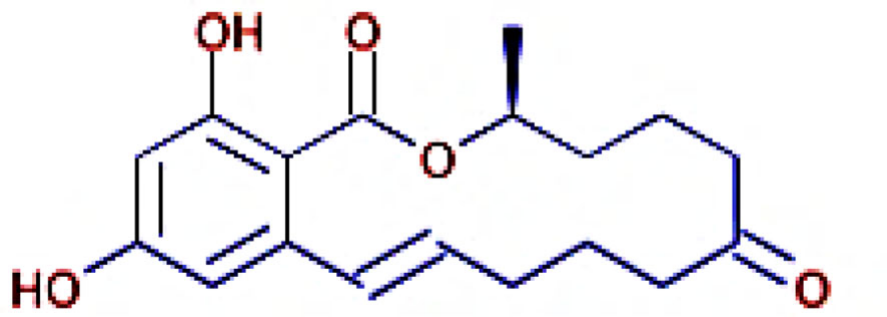
**5**	Silibinin	−5.4	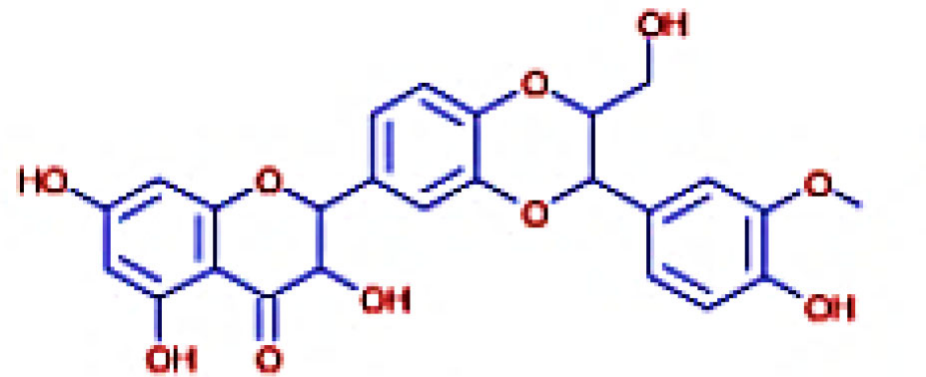
**6**	17β-estradiol	−4.4	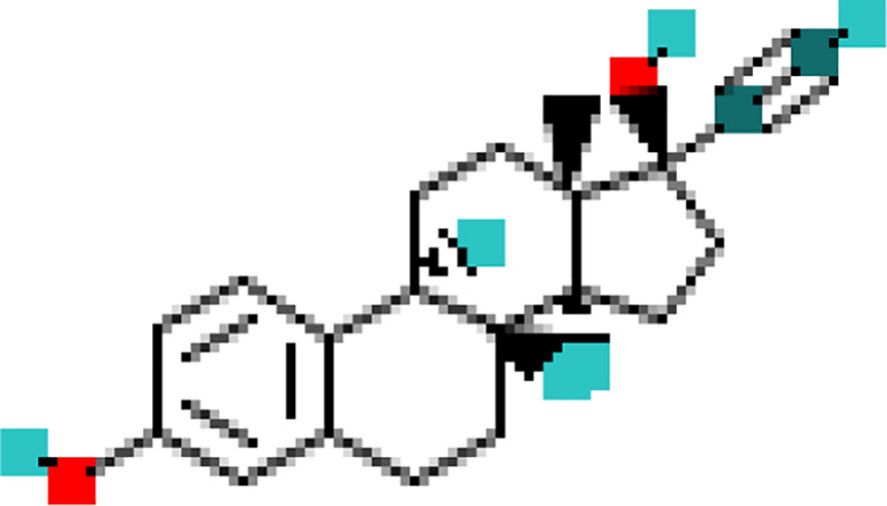

#### 2.8.2 Molecular docking

The interaction of various phytocompounds with EGFR cancer target was investigated using molecular docking studies. The compounds were evaluated based on their binding free energies, binding affinity, and ability to form hydrogen bonds and exhibit high hydrophobic interactions. To understand the protein–ligand complex behavior, the docking studies were performed using PyRx v0.8 (https://pyrx.sourceforge.io/), an open-access software (20, 38–40). The grid box was designed for the selected binding site residues to provide the best trajectory with the highest binding affinity values, shielding the active binding site residues for the selected binding site residues (41).

#### 2.8.3 Molecular dynamics simulation studies

In this study, the stability of the conformers was evaluated by performing MD simulation analysis on ligands that showed the best binding energies according to the docking and cross-docking results. Traditional MD simulations of each system were performed using the GROMACS v5.1.4 program and the CHARMM36 all-atom force field, and the topology of the phytocompounds was created by the SwissParam website. The intermolecular complex from docking experiments, which serves as the starting structure for each system, was used in the independent MD simulations. The TIP3P water model was used to neutralize each system with counter-ions in a dodecahedron box, and then the steepest descent algorithm was used to minimize energy with a force below 1,000 kJ/mol. At a temperature of 300 K, the initial equilibration was carried out for 100 ns at constant volume (NVT) and at a constant pressure (NPT) of 1 bar. The Leapfrog method was used to produce production runs of 100 ns in the NPT ensemble at an integration time of 0.2 fs since we are refining the intermolecular complex from docking investigations. Root-mean-square deviation (RMSD) values, root-mean-square fluctuation (RMSF) values, solvent-accessible surface area (SASA), and H-bond interactions were calculated from MD trajectories.

#### 2.8.4 Binding energy calculations

The findings of the MDS run for each target protein complexed with saccharopine and 17β-estradiol were used to calculate the binding free energies using the Molecular Mechanics/Poisson-Boltzmann Surface Area (MM-PBSA) method. Again, thermodynamics and molecular dynamics simulations were performed to determine the strength of the ligand interaction with the protein. The MmPbStat.py script, which uses the GROMACS 2018.1 trajectories as input, and the mmpbsa program were used to determine the binding free energy for each ligand–protein pair. The mmpbsa program uses three separate variables to calculate the binding free energy: molecular mechanical energy, polar and a polar solvation energy, and molecular mechanical energy. The calculation is finished using MDS. The most recent 100 ns of trajectory were used to compute G with dt 1,000 frames. This was evaluated using molecular mechanical energy, polar, and polar solvation energy. Equations 1 and 2 used to calculate the free binding energy are listed below.


(1) $$\Delta G_{Binding}=G_{Complex}-\left(G_{Protein}+G_{Ligand}\right)$$

$$\Delta G=\Delta E_{MM}+\Delta G_{Solvation}-T\Delta S$$

(2) $$\Delta E_{\left(Bonded+nonbonded\right)}+\Delta G_{\left(Polar+nonpolar\right)}–T\Delta SG$$

Binding: binding free energy; G_Complex_: total free energy of the protein–ligand complex; G_Protein_ and G_Ligand_: total free energies of the isolated protein and ligand in solvent, respectively; ΔG: standard free energy; ΔEMM: average molecular mechanics potential energy in vacuum; ΔG_Solvation_: solvation energy; ΔE: total energy of bonded as well as non-bonded interactions; ΔS: change in entropy of the system upon ligand binding; T: temperature in Kelvin.

## 3 Results and discussion

### 3.1 Qualitative and quantitative phytochemical screening

#### 3.1.1 Phytochemical screening

The ethanolic extract of the TCF contained flavonoids, polyphenols, tannins, alkaloids, and terpenoids, according to the results of the phytochemical screening (**Table 2**). These phytoconstituents are a class of compounds with diverse bioactivities, including anti-diabetic, anticancer, anti-inflammatory, antibacterial, and hepatoprotective effects. Additionally, research on the antioxidants found in plant extracts was done to create the goal of creating novel functional foods and nutraceuticals.

**Table 2:** Phytochemical assay results of TCF.

Sl. No.	Phytochemical test	Plant sample (ethanolic extract) TCF
1	Alkaloids	**+**
2	Carbohydrates	**+**
3	Tannins	**+**
4	Terpenoids	**+**
5	Glycosides	**+**
6	Steroids	−
7	Saponin	**+**
8	Flavanoids	**+**
9	Proteins	−
10	Mucilage	−
11	Volatile oil	−

+ means present; − means absent.

#### 3.1.2 DPPH radical scavenging activity

**Figure 2:**
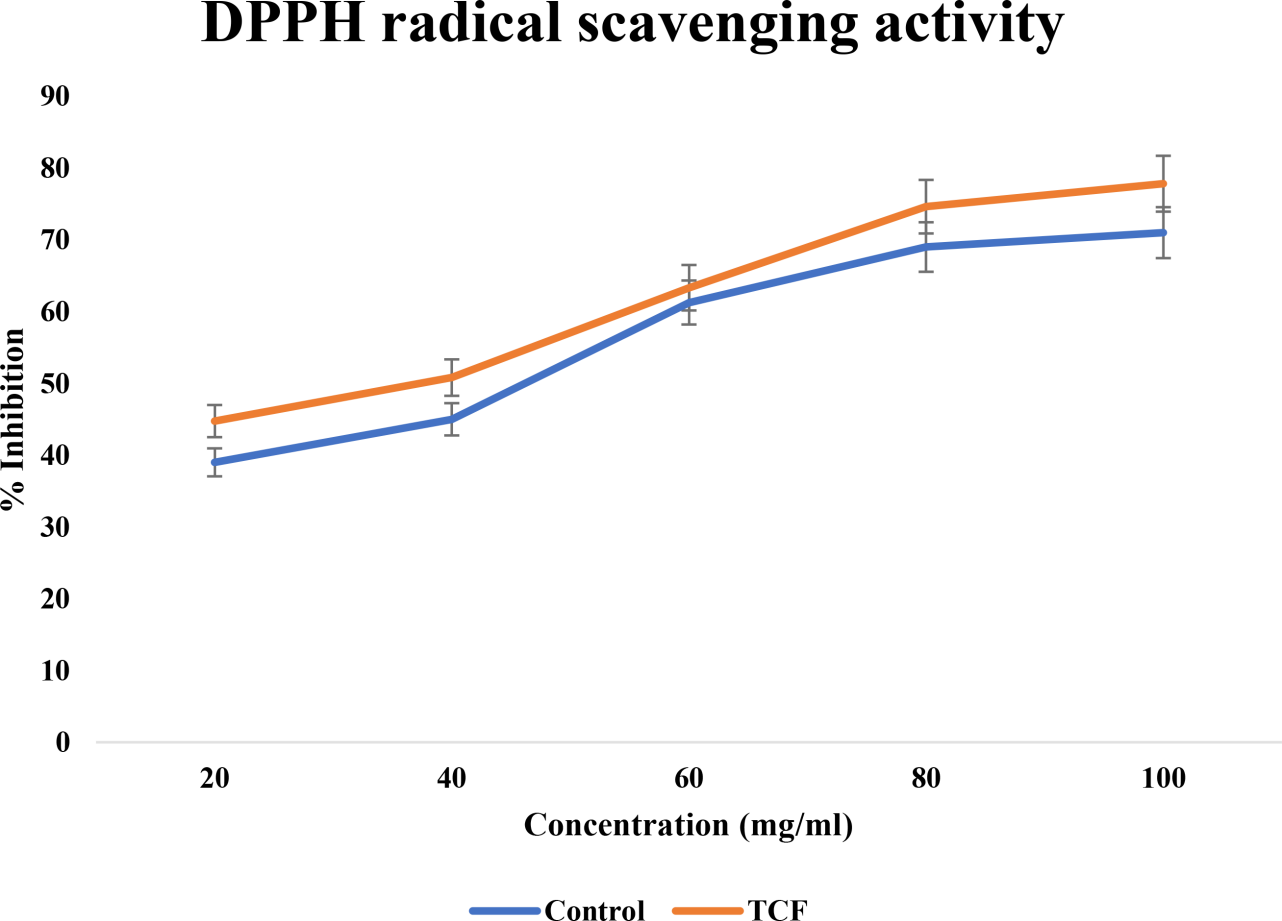
DPPH radical scavenging activity of TCF (sample) and gallic acid (control).

TCF exhibited significant movement in the current study DPPH scavenging, reaching up to 78% at 100 μg/ml concentration. When compared to gallic acid, **Figure 2** shows the dose–response curve for the DPPH scavenging activity of TCF. TCF had an IC_50_ of 44.54 ± 0.36 μg/ml while gallic acid standard had an IC_50_ of 68.47 ± 0.81 μg/ml. One of the frequently employed crucial methods to examine the existence of antioxidant molecules in plants and plant-based substances is the DPPH scavenging test. The findings showed that when compared to the standard, the TCF had potential DPPH scavenging activity.

#### 3.1.3 ABTS radical scavenging activity

**Figure 3:**
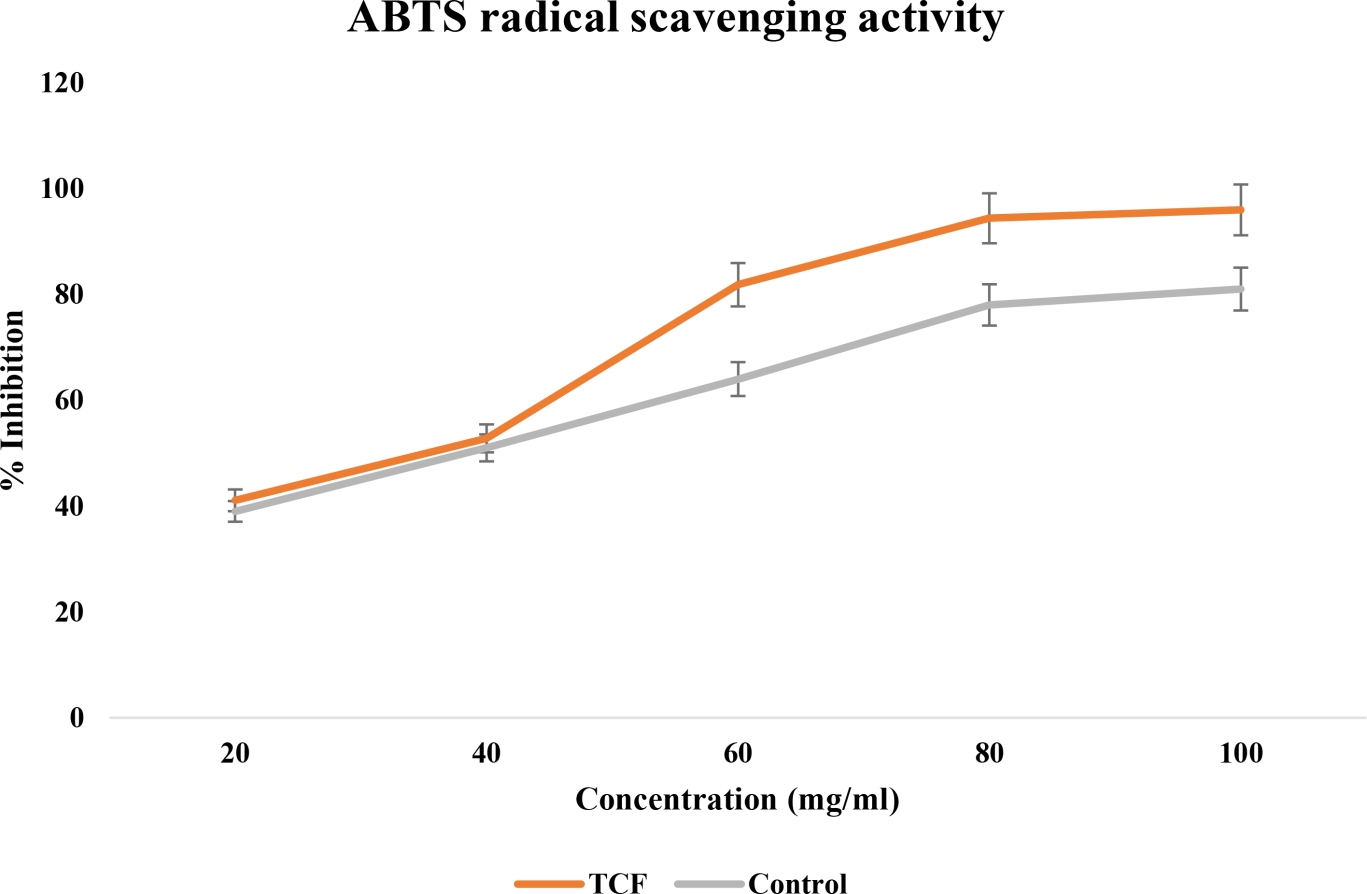
ABTS radical scavenging activity of TCF (sample) and ascorbic acid (control).

The ascorbic acid standard verified that the TCF extract was a quick and effective ABTS scavenger (**Figure 3**). With an IC_50_ value of 69.73 ± 0.70 μg/ml, it demonstrated a significant scavenging activity against ABTS radicals. It had a powerful scavenging action that was significantly comparable to ascorbic acid (81.87 ± 0.15 μg/ml). TCF had an ABTS scavenging activity percentage of 96% at a dosage of 100 μg/ml, whereas ascorbic acid had a scavenging activity percentage of 81% at the same dose.

#### 3.1.4 MTT cell cytotoxicity assay

There are various assays to measure cell viability, proliferation, and differentiation. MTT assay is one of the colorimetric, cell-based assays and is a reliable approach to quantitatively determine cell viability. Viable cells are intact and have the ability to reduce the soluble, yellow tetrazolium MTT (3-(4,5-dimethylthiazolyl-2)-2,5-diphenyltetrazolium bromide) into insoluble purple-colored formazan with the help of active mitochondrial dehydrogenase enzyme. The reaction involves reduced equivalents like NADH and NADPH as co-factors. As the formazan is insoluble, it is solubilized by cell lysis and the color intensity is measured by a colorimeter. The cells treated by drugs do not have viable cells and, hence, do not hold active enzymes to reduce MTT, and thus, they do not exhibit much change in color whose optical density is recorded and analyzed (42).

**Figure 4:**
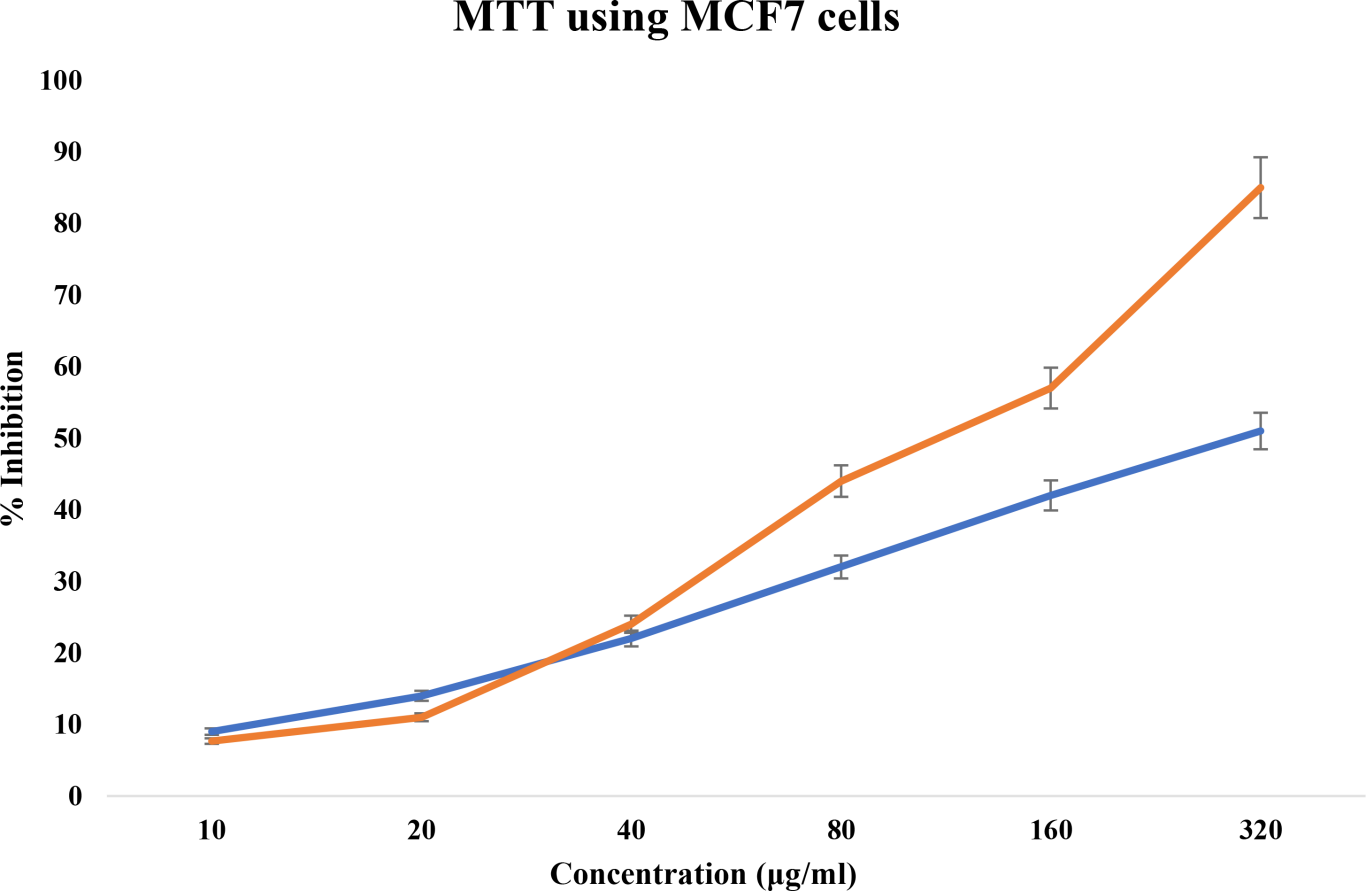
MTT cell cytotoxicity assay of TCF extract in orange and control in blue.

Different quantities of the TCF extract were introduced to the MCF-7 cell line, then incubated for 24 h to test their antiproliferative effect. According to the results of the MTT experiment, cell viability gradually declines as TCF concentration rises (**Figure 4**) with an IC_50_ of 103.2 µg/ml. Morphology of the cells was observed under an inverted microscope and was evaluated at the IC_50_ concentration of TCF.

#### 3.1.5 Hemolysis assay

**Figure 5:**
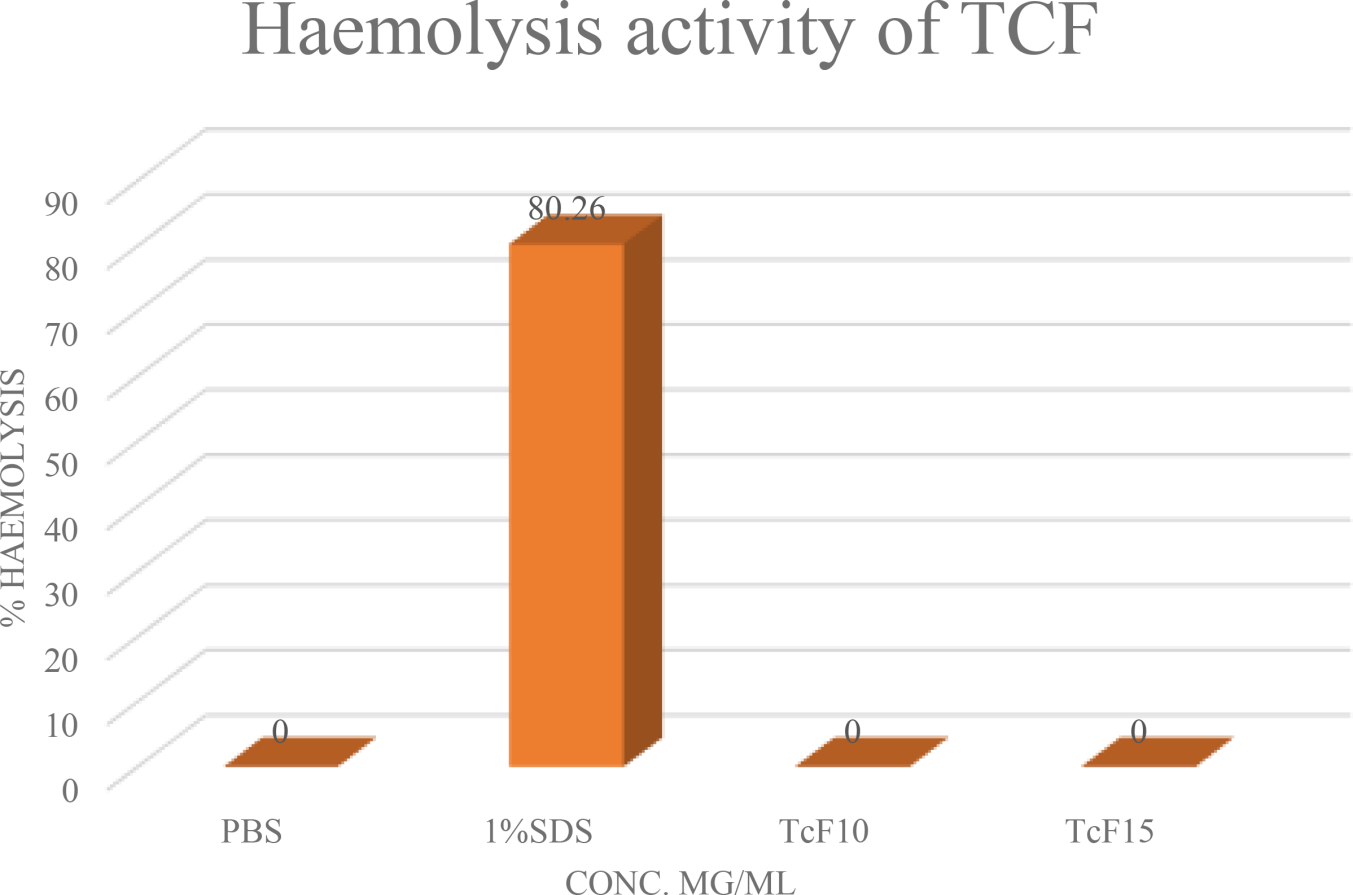
Hemolysis assay of TCF plant extract.

Plant extract might contain certain compounds that can induce lysis of erythrocytes. Such plant extracts are not reasonable to use for the treatment of cancer cells. To test the ability of plant extract to induce RBC’s lysis, hemolysis assay is performed (**Figure 5**). This test involves taking RBCs from a healthy person’s blood, treating them with the desired plant extract, and measuring the amount of hemoglobin produced as a result of lysis using a spectrophotometer (33). TCF was tested for its hemolytic abilities where no hemolysis of red blood cells was observed, which suggests that TCF plant extract samples can be used for G2M and apoptotic studies.

#### 3.1.6 G2M phase studies

When cancer cells are treated with some compound, their cell cycle progression should be inhibited in order to stop their multiplication. Cell cycle studies are to be done in order to analyze the effect of drugs on tumor cells. For the determination of cell cycle distribution and progression, flow cytometry is the most favored approach in which the DNA content of the cells is measured at a moment of the cells (43). The DNA of the cell is stained with the high-affinity DNA binding dyes. The dye used must bind to DNA stoichiometrically, which means that the cells that are in G2 phase will show twice more fluorescence as seen in the G1 phase.

**Figure 6:**
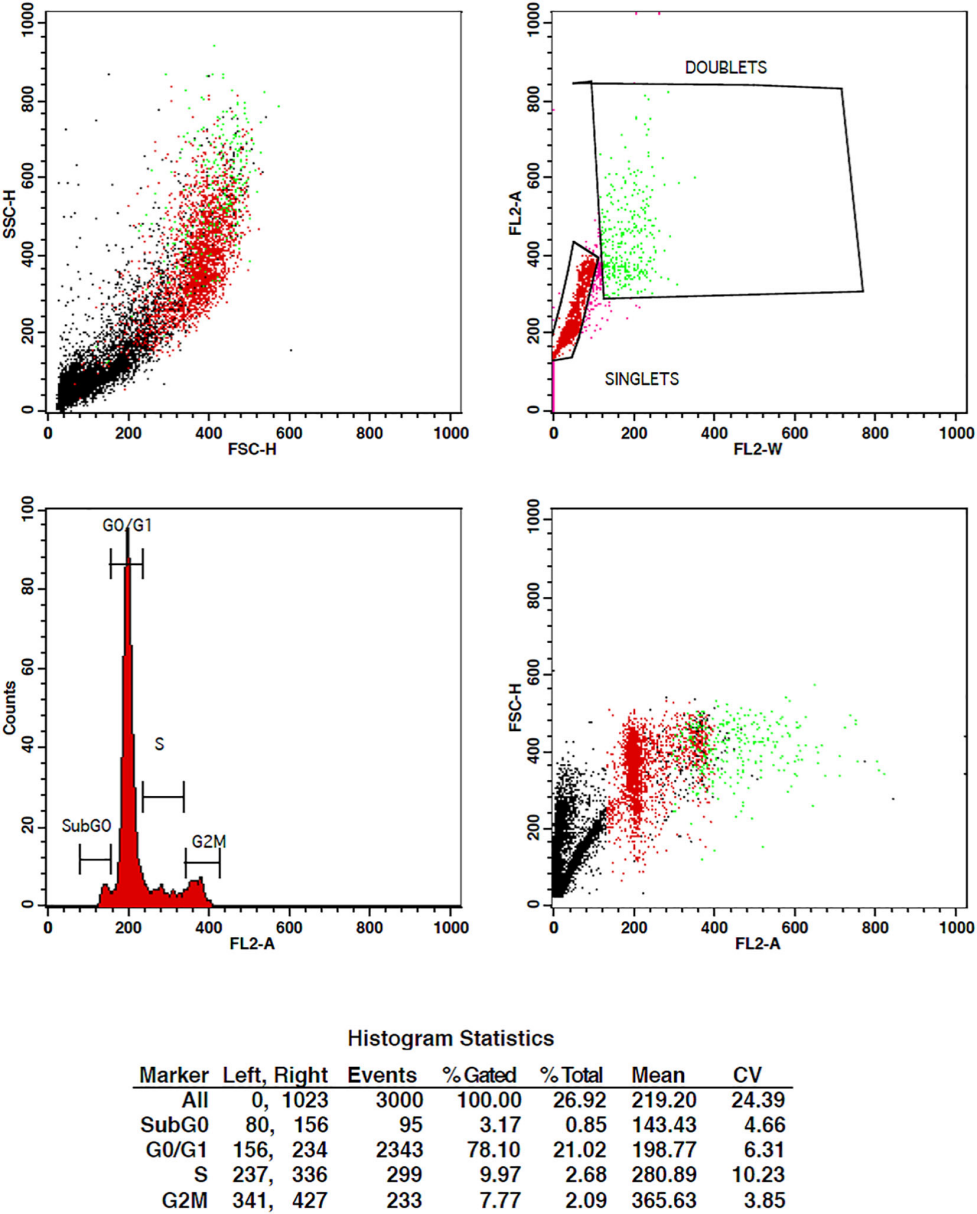
Flow cytometry plots representing the cell cycle distribution of MCF-7 cells under control conditions. The plots illustrate the percentages of cells in different phases of the cell cycle, including G0/G1, S, and G2/M. The control group shows the baseline cell cycle distribution of untreated MCF-7 cells.

**Figure 7:**
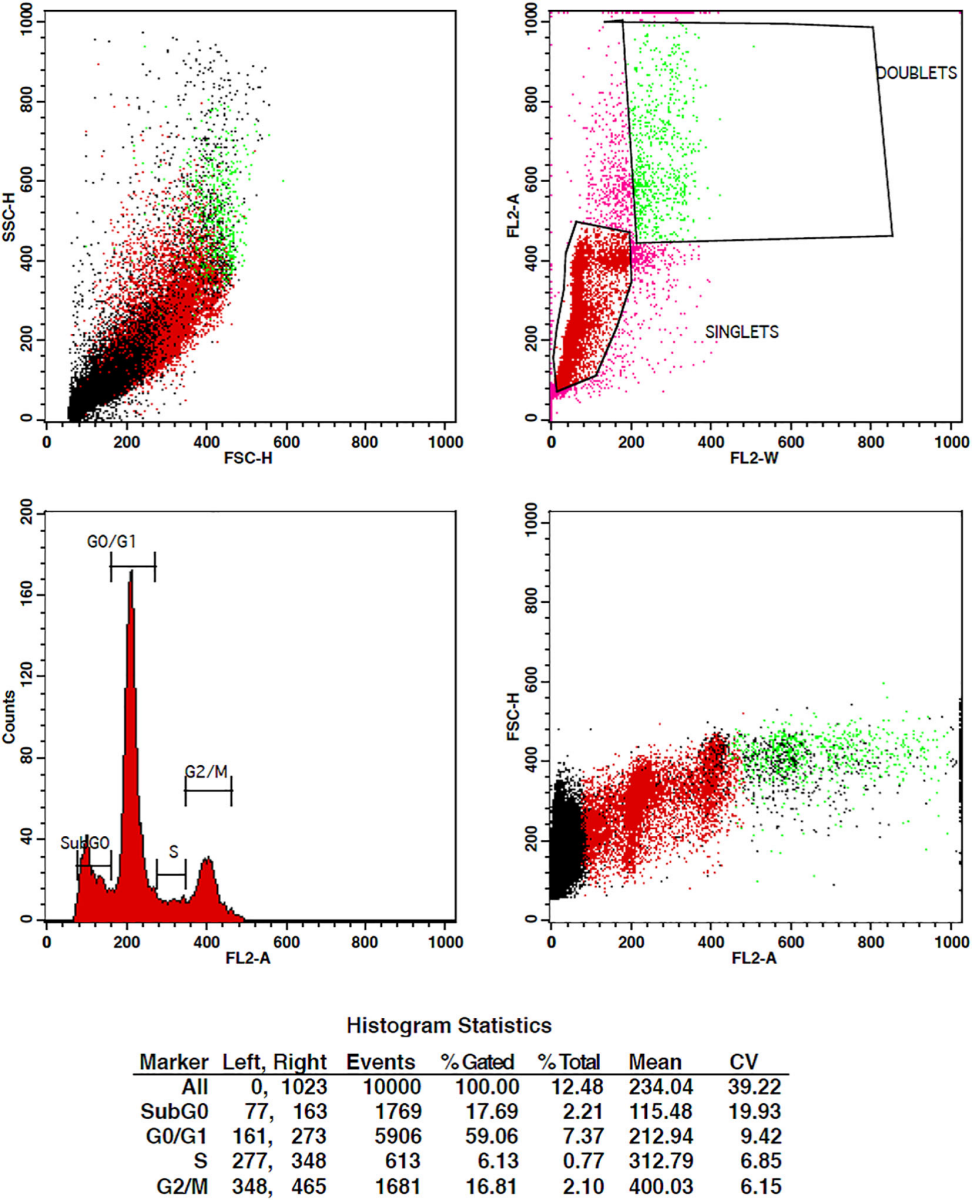
Flow cytometry plots representing the cell cycle distribution of MCF-7 cells treated with 10 µg/ml of TCF extract. The plots display the percentages of cells in the G0/G1, S, and G2/M phases of the cell cycle. Treatment with TcF extract at this concentration results in notable modifications in the cell cycle profile compared to the control group, indicating potential effects on cell cycle progression.

**Figure 8:**
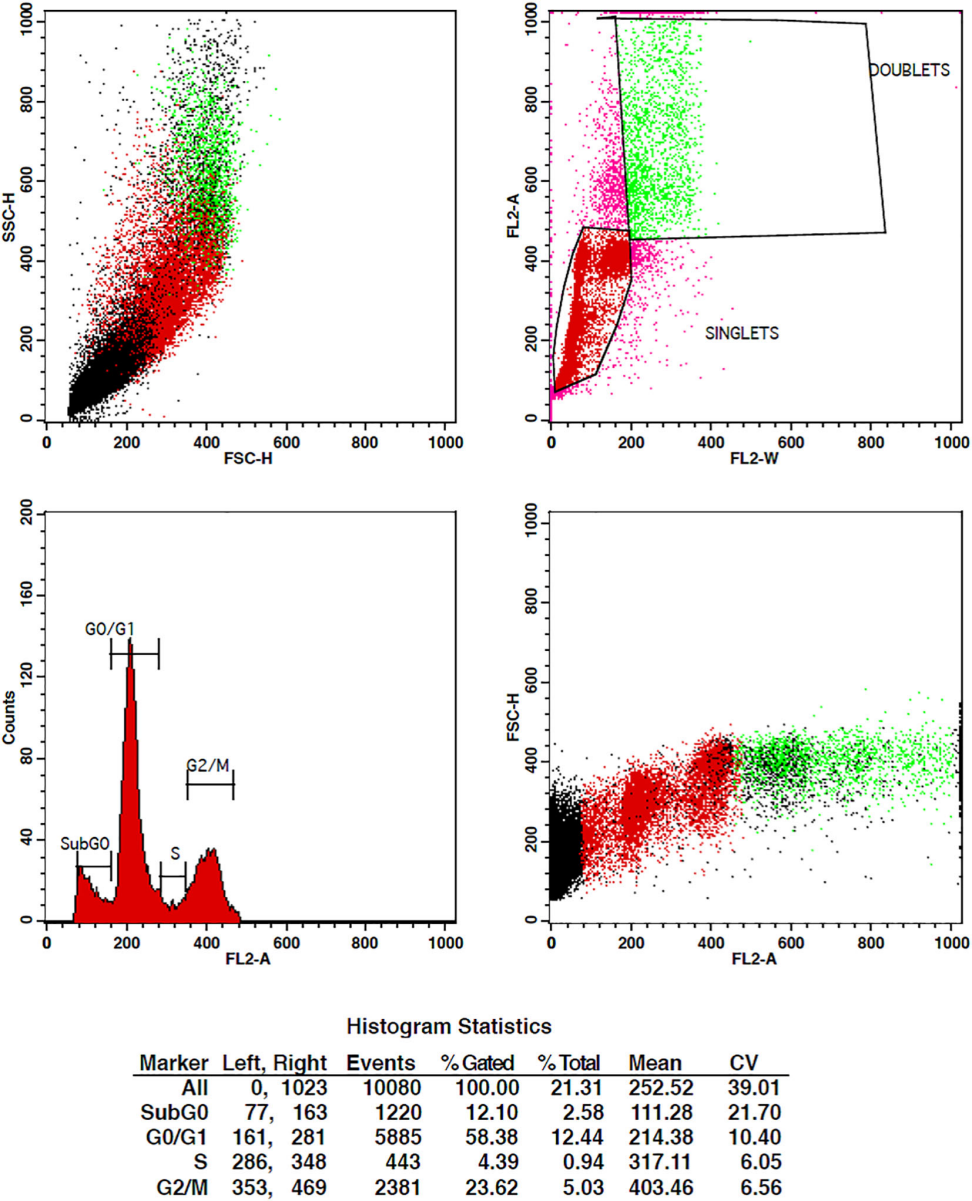
Flow cytometry plots depicting the cell cycle distribution of MCF-7 cells treated with 15 µg/ml of TcF extract. The plots illustrate the proportions of cells in the G0/G1, S, and G2/M phases of the cell cycle. Treatment with TCF extract at this concentration induces significant changes in the cell cycle profile compared to the control group, suggesting a pronounced effect on cell cycle progression.

The treatment of cells at the concentrations of 10 µg/ml and 15 µg/ml of TCF has shown G2 phase arrest of 16.81% and 23.62%, respectively, when compared to control, which has 7.7% arrest in MCF-7 cells. TCF showed 6.13% and 4.39% S phase arrest at 10 µg/ml and 15 µg/ml in MCF-7 cells, respectively (**Figures 6**–**8**). The appearance of reddish orange fluorescence with fragmented chromatin after MCF-7 cells were treated with TCF suggests that they largely induced apoptosis in MCF-7 cells. This is because EtBr, which imparts orange color, can enter only injured or dead cells.

#### 3.1.7 Apoptotic studies

Apoptosis is a type of cell death that is predetermined by the cell and is brought on by stimuli such as UV radiation and toxic substances. Under unfavorable circumstances, cells trigger the major physiological process of apoptosis, which leads to their own self-destruction. The process of stem cell proliferation and differentiation subsequently replaces these unhealthy cells with healthy ones, maintaining homeostasis. Cytotoxic medications can be used in chemotherapy because they cause apoptosis in cancer cells when they are administered to them (44).

When a cell undergoes apoptosis, several morphological changes occur in the cell. It loses its membrane integrity, starts forming blebs, cytoplasm condenses, and nuclear DNA fragmentation occurs. The plasma membrane of the cell is made of two layers of phospholipids and various proteins. These phospholipids’ bilayer arranges them as their hydrophobic tails face towards each other and hydrophilic heads face towards the extracellular fluid and cytoplasm, forming the two leaflets, outer and inner. Certain phospholipids localize themselves in any of the leaflets, e.g., phosphotidylserine is specially found in the inner leaflet of the plasma membrane. When the cell undergoes apoptosis, the phosphotidylserine becomes exposed to the outer surface of the plasma membrane, which can be used as a marker to detect whether a cell is in early apoptotic stage or not. Even the late apoptotic stages of cell can be determined by determining the DNA fragmentation in the cell, which specifically occurs in the cells that are in late apoptotic stage. Phosphatidylserine can be marked by using annexin V dye, which binds to it in the presence of Ca^+2^. Also, annexin V bound to phosphatidylserine can be detected in the flow cytometer by conjugating annexin V with chromophore FITC (green fluorescence). DNA fragmentation can be detected by using PI (red fluorescence), which binds to double-stranded DNA. If the cell shows a positive result for both dyes, then it is in the late apoptotic stage. If it is negative for both, then it is viable and if the cell shows positive only for Annexin V-FITC, then it is in the early apoptotic stage (Shivalingaiah et al., 2022).

**Figure 9:**
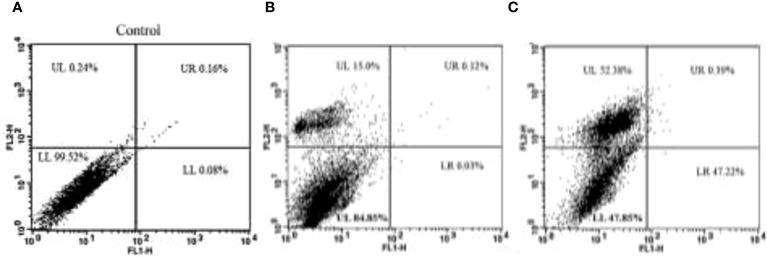
**(A)** The control plot represents MCF-7 cells in their untreated state, showing the baseline distribution of cell populations in the G0/G1, S, and G2/M phases of the cell cycle. **(B, C)** The plots of cells treated with 10 µg/ml and 15 µg/ml of TCF extract demonstrate the effects of the sample on the cell cycle profile. Treatment with TCF at both concentrations induces changes in the distribution of cells across the cell cycle phases, indicating potential alterations in cell cycle progression.

This investigation looked at how TCF affected the induction of apoptosis in MCF-7 cells (**Figure 9**). Dot-plot graphs were divided into four quadrants: the lower left quadrant displayed viable cells, the lower right quadrant showed early-phase apoptotic cells, the upper right quadrant displayed late-phase apoptotic or dead cells, and the upper right quadrant showed necrotic cells (the upper left quadrant). At 24 h, the MCF-7 lower right (LR) quadrant’s early cell populations were 0.03% and 47.22%. Additionally, at a dosage of 10 and 15 µg/ml, the percentage of late apoptotic cells in the upper right quadrant of the MCF-7 sample was 15.0% and 52.38%. After the therapy was given for an additional 72 h, more than 50% of MCF-7 cells were in the late apoptotic phase.

### 3.2 LDH assay

**Figure 10:**
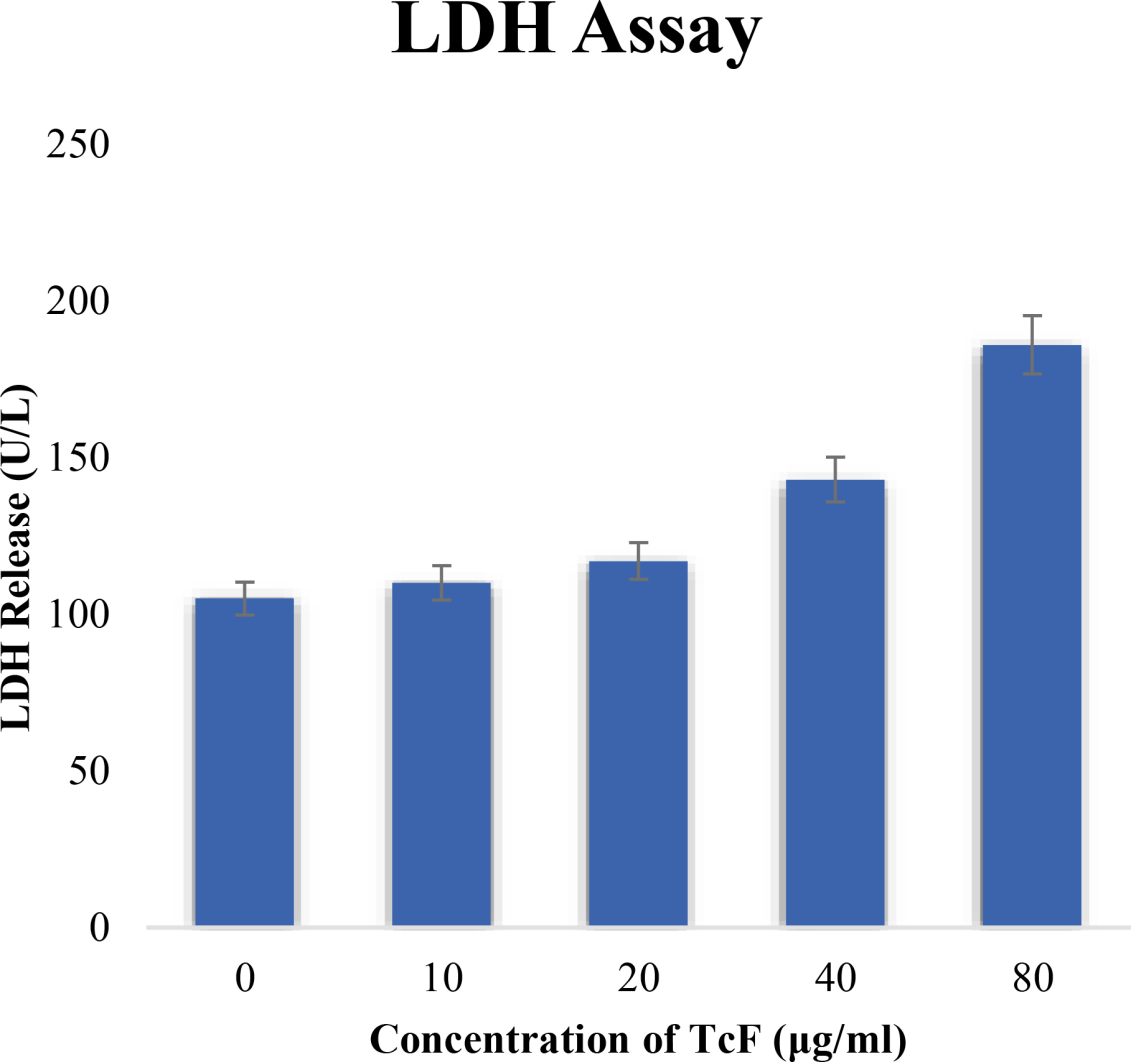
LDH activity of cultured MCF-7 cells exposed to TCF.

The LDH activity of culture MCF-7 cells exposed to TCF was measured, and no significant changes in LDH release were seen at doses less than 20 µg/ml. Cell membrane damage causes the LDH leakage assay. After the cells were treated to the anticancer drugs, LDH levels (a sign of necrosis) in the cell media clearly increased (**Figure 10**). The necrotic action of TCF at high concentrations may explain the increase in LDH activity of MCF-7 cells exposed to high doses of TCF.

### 3.3 AO/EtBr staining assay

**Figure 11:**
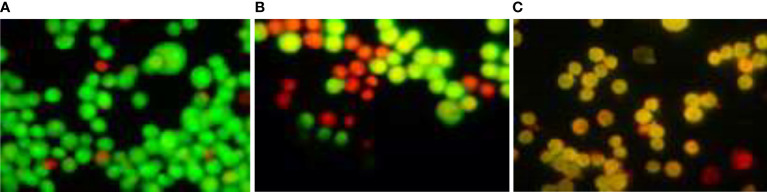
AO/EtBr staining assay. **(A)** Control, **(B)** TCF 10 µg/ml, and **(C)** TCF 15 µg/ml on the MCF7 cell line.

An increase in apoptosis-inducing cell cycle arrest was found when MCF-7 cells were treated with TCF; apoptosis-induced cell death was determined using the acridine orange/ethidium bromide dual-labeling procedure. The cells were stained with acridine orange and ethidium bromide after being treated with escalating doses of TCF for 24 h. Under a fluorescent microscope, apoptosis-inducing cells contain compacted chromatin that takes up the ethidium bromide stain and turns orange, whereas live cells appear as green-colored entities (40×). The appearance of reddish orange fluorescence with fragmented chromatin after MCF-7 cells were treated with TCF suggests that they largely induced apoptosis in MCF-7 cells. This is because EtBr, which imparts orange color, can enter only injured or dead cells as shown in **Figure 11**.

### 3.4 ROS determination

**Figure 12:**
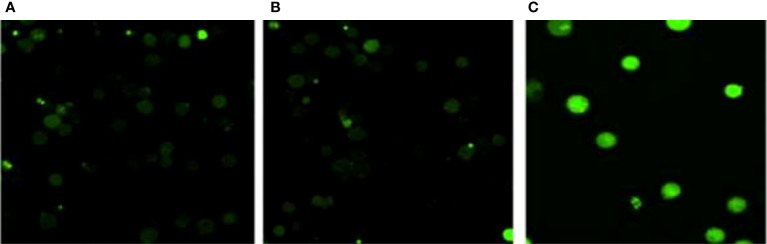
ROS determination assay. **(A)** Control, **(B)** TCF 10 µg/ml, and **(C)** TCF 15 µg/ml on the MCF7 cell line.

The formation of reactive oxygen species (ROS) in MCF-7 cells exposed to TCF was observed to rise in a concentration-dependent manner. When comparing untreated MCF-7 cells to TCF exposed MCF-7 cells, fluorescence microscopic examination clearly revealed a dose-dependent increase in the intensity of green fluorescence. The highest ROS production was seen at 15 µg/ml, when it was 2.2-fold higher than the untreated control (**Figure 12**).

### 3.5 *In silico* studies

#### 3.5.1 Molecular docking studies

Molecular docking is a computational technique used in the field of structural biology to predict the binding mode and affinity of a small molecule (ligand) to a target protein (receptor). The technique involves generating a set of possible conformations of the ligand and receptor, and then evaluating the potential interactions between them using various scoring functions. The goal is to identify the most favorable binding pose, which is the conformation of the ligand that maximizes its interactions with the receptor. Docking is widely used in drug discovery and design, as it can provide valuable insights into the binding mechanisms of potential drug candidates with their targets. It can also be used to screen large libraries of compounds to identify potential lead compounds that could be developed into drugs.

**Figure 13:**
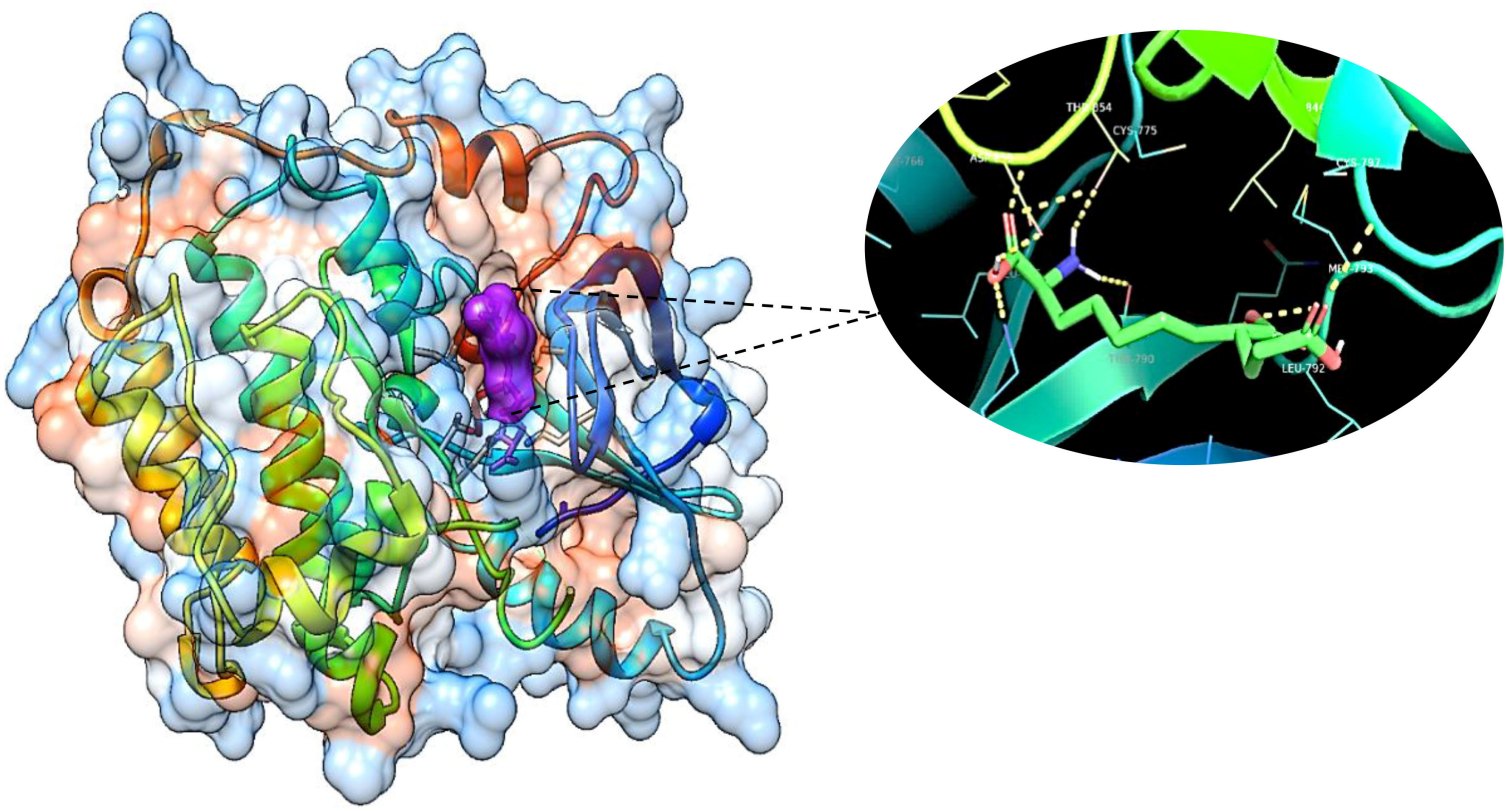
The 3D image of saccharopine (purple pocket) depicting the bonded molecular interactions (yellow dotted lines) with the 3W2S receptor (blue hydrophobic pocket).

**Figure 14:**
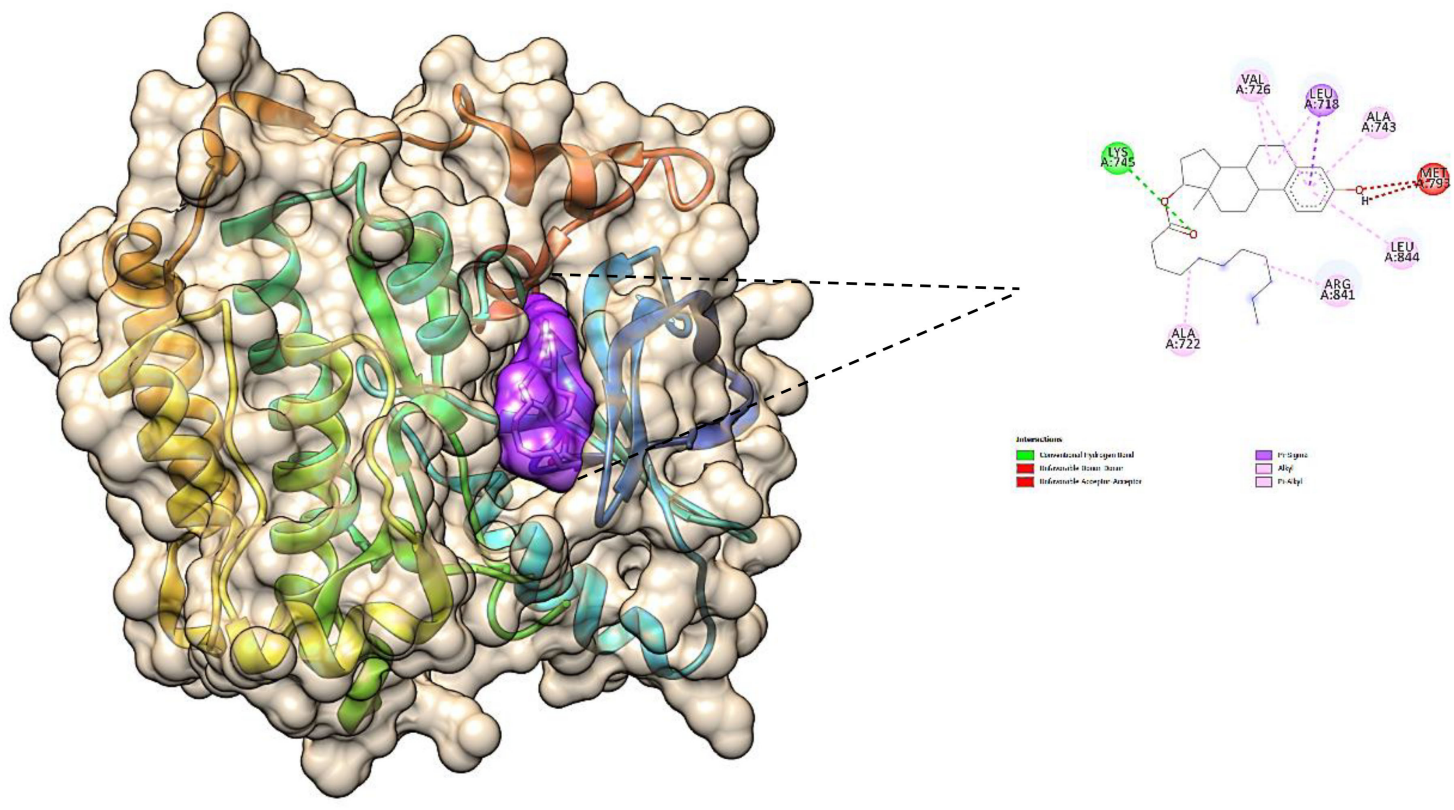
The 3D image of 17β-estradiol (purple pocket) depicting the bonded molecular interactions (yellow dotted lines) with the 3W2S receptor (brown hydrophobic pocket).

The obtained results confirm the best docking poses of all the selected ligands corresponding to their lowest binding affinity. The obtained protein–ligand interaction was further visualized and analyzed using Chimera software as it shows the binding residues that form the bonded and non-bonded interactions between the protein–ligand complex. The 3D docking interaction of breast cancer protein 3W2S with ligands saccharopine and 17β-estradiol is shown in **Figures 13** and **14**. The saccharopine ligand exhibits a least binding affinity of −9.7 kcal/mol and GLN-791, MET-793, CYS-797, THR-854, and ASP-855 residues form the bonded interactions, whereas VAL-726, ALA-743, and LEU-844 residues form the non-bonded interactions with the 3W2S receptor. 17β-Estradiol comparatively exhibits a low binding affinity of −4.4 kcal/mol and LYS-745 residue formed one-bonded interaction, whereas LEU-718, ALA-722, VAL-726, ALA-743, ARG-841, and LEU-844 residues formed the non-bonded interactions, and it was also seen that the standard drug formed a non-favorable interaction with the MET-793 residue of the 3W2S receptor. The obtained *in silico* results infer that saccharopine molecule as the most potent phytochemical present in TCF leaf can be used as the best breast cancer inhibitor compared to the standard, as it showed very high binding affinity and very low binding energy with a good amount of bonded and non-bonded interactions.

#### 3.5.2 Molecular dynamics simulation studies

Molecular dynamics simulation (MDS) is a computational method used to investigate the dynamic behavior of biological macromolecules such as proteins and nucleic acids. MDS involves the application of classical mechanics equations to simulate the movements of atoms and molecules over time. It can provide valuable information on the conformational changes, flexibility, and stability of macromolecular systems in solution or at interfaces. By applying external forces and thermal fluctuations to the simulated system, MDS can mimic the behavior of biological systems in a realistic manner. MDS is widely used in drug discovery, protein engineering, and materials science to predict the behavior of molecules and optimize their properties. MDS is commonly used to predict and refine the binding of small molecules with proteins, as well as to assess the stability of these interactions. In this study, MD simulations were utilized to improve the predicted binding of screened phytocompounds of TCF with their individual drug targets, as well as to examine the intermolecular interactions and stability of these compounds with their targets, with the aim of discovering potential anti-lung cancer lead molecules. The predicted complexes from docking studies were used as the initial structures for the MD simulations.

To analyze the conformational fluctuations of protein–ligand complexes, this study utilized RMSD calculations. The RMSD measures the deviation of the protein backbone and ligand coordinates from their initial docked pose coordinates. It calculates the average distance between superimposed protein and ligand structures over time, and it is a valuable metric to assess the stability of the complex. The RMSD plots were constructed using qt-Grace software and showed that the RMSD values of protein (3W2S) along with saccharopine remained stable within the range of 0.13 to 0.23 Å from 0- to 20-ns frames, and then slightly fluctuated from 70 to 85 ns in the complex. However, the 3W2S–17β-estradiol complex deviated throughout the simulation, making it an unstable molecule.

RMSF was also used to examine the regions of the protein that deviate the most/least from the mean. It calculates the root mean square distances with respect to the central axis of rotation. The RMSF plots generated from this study showed that the complexes were stable, with fluctuations only at the terminal ends and loop regions. The RMSF plots of 3W2S with saccharopine indicated that all amino acids located in the enzyme’s active site had RMSF fluctuations between 0.01 and 0.35Å, indicating that the studied compounds maintained close contact with their binding pockets during the MD simulations. However, 3W2S with 17β-estradiol showed higher variations compared to saccharopine compound.

**Figure 15:**
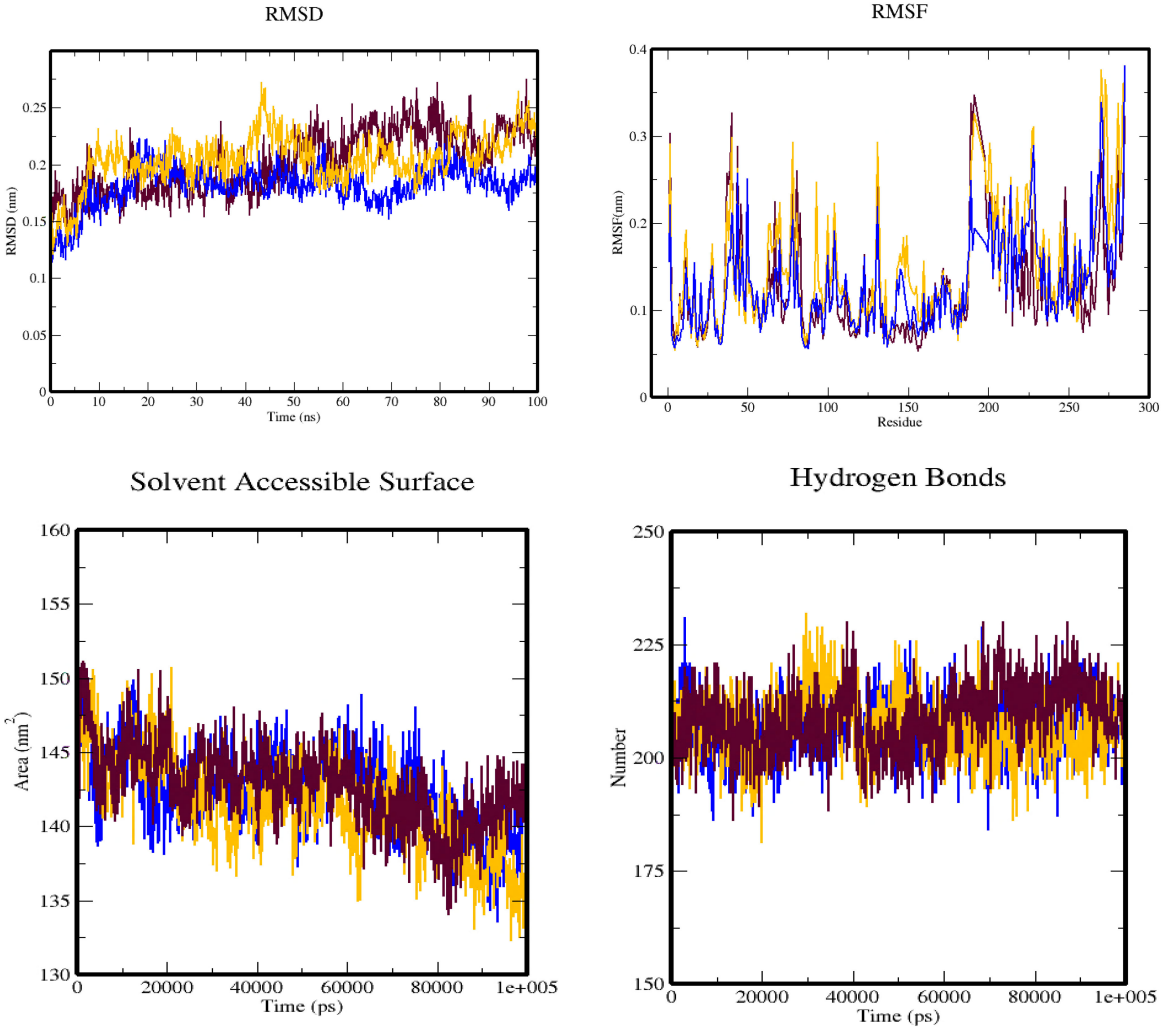
The simulation trajectories obtained from the molecular dynamics simulation over 100 ns depict four plots: **(A)** RMSD plot, **(B)** RMSF plot, **(C)** SASA plot, and **(D)** hydrogen bond plot. These plots are color-coded, with maroon representing the 3W2S alone, blue representing the 3W2S–saccharopine complex, and orange representing the 3W2S–17β-estradiol complex.

To gain a better understanding of the changes in the complex’s surface area, the SASA of the simulation complex was examined. A higher SASA suggests an increase in surface volume, while a lower SASA implies a reduction in the complex’s size. The SASA measures the surface area of the hydrophobic core created by protein–ligand interactions. Notably, the 3W2S protein–saccharopine complex exhibited consistent SASA values compared to other complex (**Figure 15**).

Additionally, hydrogen bonds were evaluated to determine the bonding and structural changes in the complex. The stability of the complex was determined by the hydrogen bonds present during the simulation. The hydrogen bonds were evaluated across the simulation duration, and the entire complex exhibited a stable trend. Hydrogen bonding is a crucial factor in molecular recognition specificity, and the hydrogen bond patterns of the complexes were investigated over the entire 100-ns simulation trajectory using GROMACS. The results showed that 3W2S with saccharopine formed more hydrogen bonds than all other conformers, while the 3W2S plus 17β-estradiol complex formed only one hydrogen bond, along with one unfavorable bond, making it an unstable molecule compared to saccharopine.

#### 3.5.3 Binding energy calculations

Binding energy calculations are used to quantify the strength of the interactions between a ligand and a target protein. The binding energy is the amount of energy released or absorbed when the ligand and target protein interact and form a complex. It is calculated by subtracting the energy of the unbound ligand and target protein from the energy of the bound complex. A lower binding energy indicates a stronger interaction between the ligand and protein. Various energy metrics, such as van der Waals energy, electrostatic energy, polar solvation energy, SASA energy, and binding energy, can be utilized to measure the extent of ligand–target protein binding interactions during molecular dynamics simulations. These calculations can help in the design and development of new drugs by identifying compounds that have high binding affinity and stability with the target protein.

In molecular dynamics simulations, several energy metrics such as van der Waals, electrostatic, polar solvation, SASA, and binding energies are commonly used to quantify the extent of ligand–target protein binding interactions. In this study, the protein–ligand combination was primarily constructed using the electrostatic energy metric, followed by van der Waals energy, SASA energy, and binding energy. However, the polar solvation energy was not considered for protein–ligand complex formation since its values appeared to be positive. The 3W2S complex, which was formed by saccharopine and 17β-estradiol, exhibited the highest binding affinity and was therefore chosen for the binding energy calculation studies. Moreover, the standard deviations of the protein–ligand complex were computed. A lower standard deviation suggests that the data values are close to the mean, while a higher standard deviation implies that the data values are dispersed over a broader range. However, in the complex, no high standard deviations were observed, indicating that saccharopine and 17β-estradiol bind to the protein with a high binding affinity and stable interaction. The binding free energy calculations of the protein–ligand complex are summarized in **Table 3**.

**Table 3:** Binding free energy calculations of EGFR tyrosine kinase complexed with the lead metabolite of *Trichoderma* spp. and standard inhibitor.

Category	3W2S–saccharopine complex	3W2S–17β-estradiol complex
	Values (kcal/mol)	Values (kcal/mol)
**Electrostatic energy**	−133.567	−95.788
**Van der Waals energy**	−5.682	−3.564
**Polar salvation energy**	17.164	26.810
**SASA energy**	−10.240	−2.545
**Binding energy**	−87.740	−18.338

## 4 Conclusion and future prospects

As one of the top causes of death for women, breast cancer must be detected and treated as soon as possible in order to avoid metastasizing. Plant-derived bioactive chemicals have the potential to be powerful medicines; however, many plants have not yet been studied for their apoptogenic and antiproliferative effects. According to the results of the current study, *T. chebula* fruit extract has been found to be non-hemolytic and cytotoxic to the MCF-7 breast cancer cell line. As a result, the bioactive chemicals from the fruit could be exploited as a possible therapeutic agent against breast cancer. The investigation of saccharopine as a potential anticancer agent necessitates more comprehensive *in vivo* studies to assess its efficacy, safety, and therapeutic potential. Although encouraging outcomes have been observed in *in vitro* experiments, *in vivo* studies are essential for evaluating the compound’s effectiveness in a complex biological system and assessing its pharmacokinetics, toxicity profile, and long-term effects. However, the isolation and testing of saccharopine pose challenges due to its limited natural occurrence and availability. Obtaining sufficient quantities of pure saccharopine for extensive *in vivo* studies may require specialized synthetic or biosynthetic approaches. Moreover, the administration and delivery of saccharopine *in vivo* present additional obstacles, such as determining the optimal dosage, route of administration, and formulation to achieve systemic distribution and tumor targeting. Additionally, understanding saccharopine’s selectivity, potency, and potential limitations in its anticancer efficacy, as well as its impact on different cancer types, heterogeneity, and underlying mechanisms of action, is crucial for its further development as an anticancer therapeutic. Overall, addressing these challenges and conducting thorough *in vivo* investigations will provide valuable insights into the therapeutic potential of saccharopine and guide future preclinical and clinical studies.

The goal of the current study was to investigate and assess the efficacy of fruit in inhibiting the estrogen receptor and progesterone receptor-positive MCF-7 cell line. In preliminary antioxidant and anti-inflammatory studies, phytochemicals with anticancer potential were found. Via the induction of necrosis and cell cycle arrest, TCF extract reliably decreased MCF-7 breast cancer cell survival. In cytotoxicity testing, the phytocompounds identified in TCF were found to kill cells. The cytometric results provided strong support for the pro-apoptotic compulsions of G0/G1 phase cell cycle arrest that were responsible for the observed cytotoxicity. Based on *in silico* observations, it may be hypothesized that the MCF-7 cytotoxicity of TCF occurs via the involvement of the EGFR receptor.

## Data availability statement

The datasets for this article are not publicly available due to concerns regarding participant anonymity. Requests to access the datasets should be directed to the corresponding author.

## Author contributions

Conceptualization: PR and SP. Methodology: PR, SP, RA, CD, CSS, and SK. Software: SP. Validation: PR, SP, SM, and GS. Formal analysis: PR, SP, AS, MA, SE, and CS. Investigation: PR and SP. Data curation: PR, DG, ES, VS, NM, and SP. Writing—original draft preparation: PR, SP, SC, and CS. Writing—review and editing: PR, SK, and CD. Visualization: SP. All authors listed have made a substantial, direct, and intellectual contribution to the work, and approved it for publication.
