# Supplementary material for: Cell cycle arrest and apoptotic studies of Terminalia chebula against MCF-7 breast cancer cell line: an in vitro and in silico approach
Source: Front Oncol. 2023 Aug 29;13:1221275. doi: 10.3389/fonc.2023.1221275 (PMC10497218; doi:10.3389/fonc.2023.1221275)
Supplement: Supplementary file 1 [file DataSheet_1.docx]

**Supplementary data**

1. **DPPH radical scavenging activity**

**Table S1:** Represents the triplicate results obtained for DPPH assay.

| **Concentration (mg/ml)** | **% Inhibition** | | | **Average** |
| --- | --- | --- | --- | --- |
|  | **Trial 1** | **Trial 2** | **Trial 3** |  |
| 20 | 45.02+ 0.46 | 45.0+ 0.18 | 44.22+ 0.35 | 44.75 |
| 40 | 51.37+ 0.11 | 51.05+ 0.15 | 50.0+ 0.94 | 50.81 |
| 60 | 64+ 0.10 | 63.99+ 0.51 | 62.01+ 0.50 | 63.33 |
| 80 | 74.99+ 0.57 | 74.80+ 0.27 | 74.08+ 0.38 | 74.62 |
| 100 | 78.45+ 0.66 | 78.24+ 0.52 | 76.77+ 0.38 | 77.82 |

1. **ABTS radical scavenging activity**

**Table S2:** Represents the triplicate results obtained for ABTS assay.

| **Concentration (mg/ml)** | **% Inhibition** | | | **Average** |
| --- | --- | --- | --- | --- |
|  | **Trial 1** | **Trial 2** | **Trial 3** |  |
| 20 | 41.43+ 0.39 | 41.21+ 0.09 | 40.63+ 0.16 | 41.09 |
| 40 | 53.53+ 0.44 | 53.50+ 0.07 | 51.36+ 0.45 | 52.80 |
| 60 | 82.69+ 0.40 | 82.26+ 0.29 | 80.51+ 0.20 | 81.82 |
| 80 | 94.6+ 0.21 | 94.5+ 0.67 | 94.045 + 0.35 | 94.37 |
| 100 | 96.47+ 0.08 | 96.01+ 0.89 | 95.40+ 0.53 | 95.96 |

1. **MTT cell cytotoxicity assay**

**Table S3:** Represents the triplicate results obtained for MTT assay.

| **Concentration (mg/ml)** | **% Inhibition** | | | **Average** |
| --- | --- | --- | --- | --- |
|  | **Trial 1** | **Trial 2** | **Trial 3** |  |
| 0 | 0 | 0 | 0 | 0 |
| 10 | 8 | 7 | 7 | 7.67 |
| 20 | 12 | 10 | 11 | 11 |
| 40 | 24 | 23 | 20 | 24 |
| 80 | 45 | 40 | 38 | 44 |
| 160 | 58 | 56 | 51 | 55 |
| 320 | 86 | 82 | 84 | 84 |

**Purification and characterization of Saccharopine compound from TCF extract**


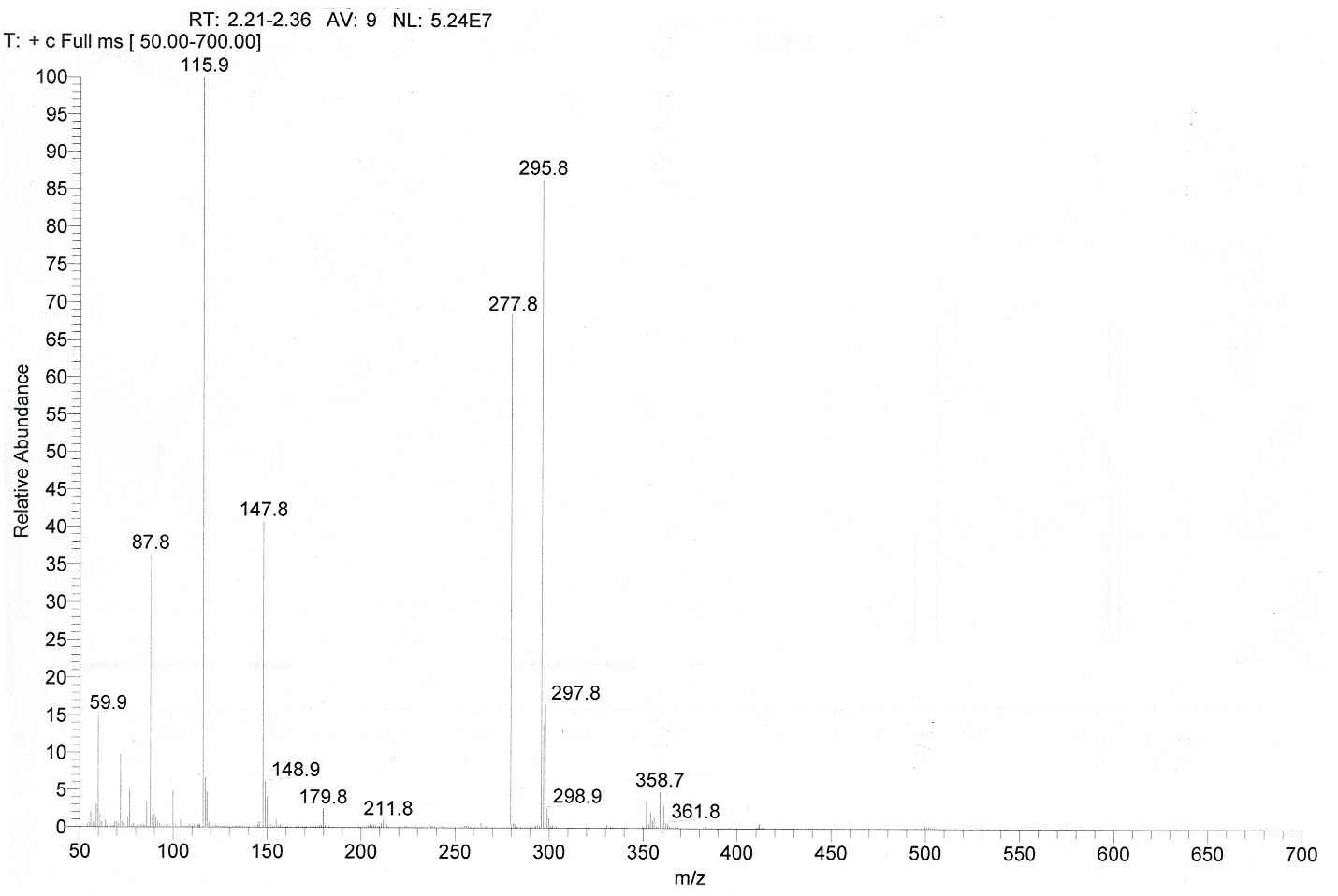


**Figure S1: Mass spectrum of isolated Saccharopine molecule.**


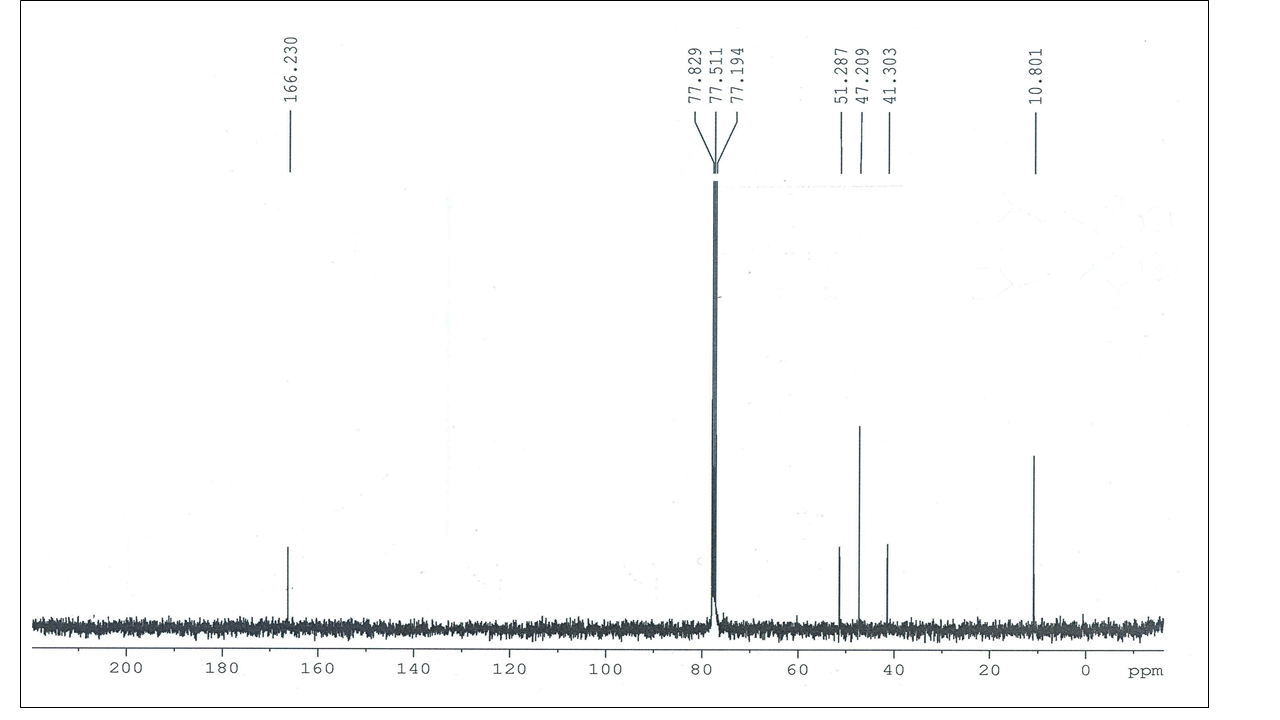


**Figure S2: ^13^C-NMR spectrum of isolated Saccharopine molecule.**


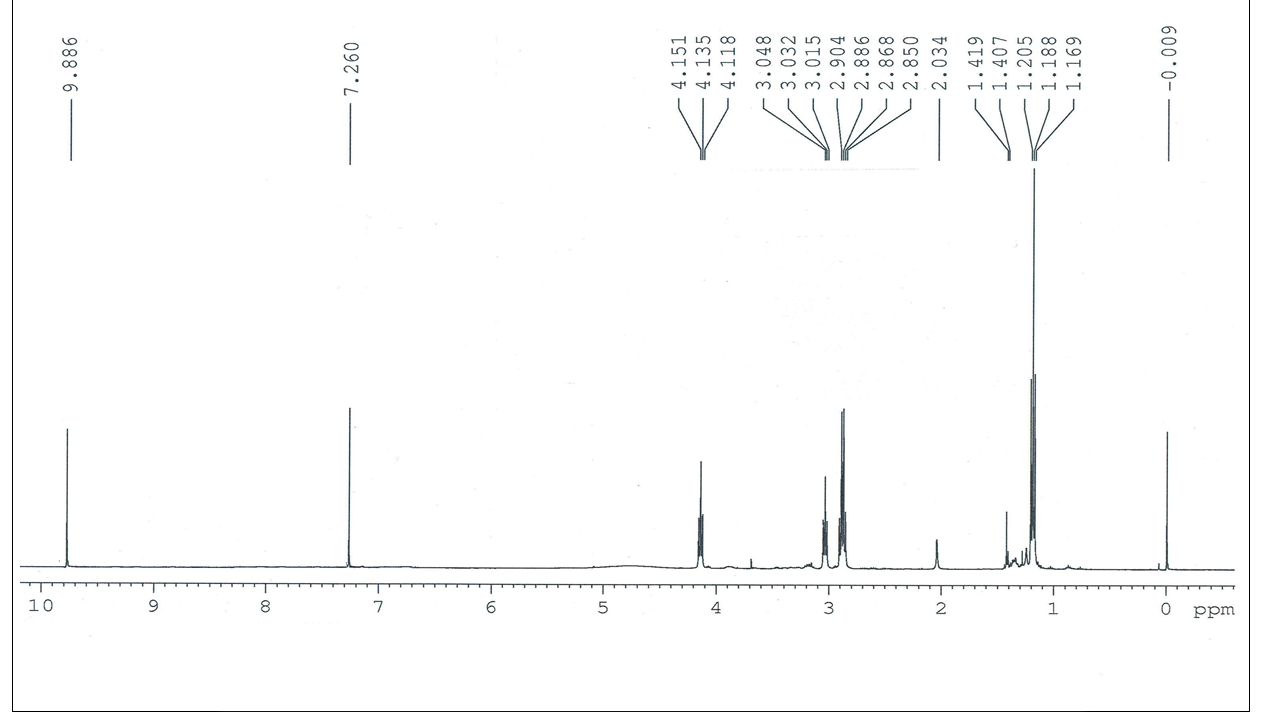


**Figure S3: ^1^H-NMR spectrum of isolated Saccharopine molecule.**
